# Rice Genome-Scale Network Integration Reveals Transcriptional Regulators of Grass Cell Wall Synthesis

**DOI:** 10.3389/fpls.2019.01275

**Published:** 2019-10-18

**Authors:** Kangmei Zhao, Fan Lin, Sandra P. Romero-Gamboa, Prasenjit Saha, Hyung-Jung Goh, Gynheung An, Ki-Hong Jung, Samuel P. Hazen, Laura E. Bartley

**Affiliations:** ^1^Department of Microbiology and Plant Biology, University of Oklahoma, Norman, OK, United States; ^2^Department of Biology, University of Massachusetts, Amherst, MA, United States; ^3^Graduate School of Biotechnology & Crop Biotech Institute, Kyung Hee University, Yongin, South Korea

**Keywords:** Network, cell wall, transcription factor, hydroxycinnamate, comparative analysis, regulatory evolution

## Abstract

Grasses have evolved distinct cell wall composition and patterning relative to dicotyledonous plants. However, despite the importance of this plant family, transcriptional regulation of its cell wall biosynthesis is poorly understood. To identify grass cell wall-associated transcription factors, we constructed the Rice Combined mutual Ranked Network (RCRN). The RCRN covers >90% of annotated rice (*Oryza sativa*) genes, is high quality, and includes most grass-specific cell wall genes, such as mixed-linkage glucan synthases and hydroxycinnamoyl acyltransferases. Comparing the RCRN and an equivalent *Arabidopsis* network suggests that grass orthologs of most genetically verified eudicot cell wall regulators also control this process in grasses, but some transcription factors vary significantly in network connectivity between these divergent species. Reverse genetics, yeast-one-hybrid, and protoplast-based assays reveal that OsMYB61a activates a grass-specific acyltransferase promoter, which confirms network predictions and supports grass-specific cell wall synthesis genes being incorporated into conserved regulatory circuits. In addition, 10 of 15 tested transcription factors, including six novel Wall-Associated regulators (WAP1, WACH1, WAHL1, WADH1, OsMYB13a, and OsMYB13b), alter abundance of cell wall-related transcripts when transiently expressed. The results highlight the quality of the RCRN for examining rice biology, provide insight into the evolution of cell wall regulation, and identify network nodes and edges that are possible leads for improving cell wall composition.

## Introduction

Cultivated grasses are the most abundant sustainable biomass source produced worldwide (Lal, 2005), and cell walls constitute the bulk of plant dry mass available for conversion to biofuels and other bioproducts. In vascular plants, primary walls surround growing cells; whereas, after cessation of growth, secondary walls form around cells such as tracheids, vessels, and fibers. Primary and secondary cell walls consist of both conserved components and those that vary across plant diversity (Liepman et al., 2010; Popper et al., 2011; Fangel et al., 2012). The major vascular plant cell wall components are cellulose, lignin, and matrix polysaccharides, including hemicelluloses and pectins. Cellulose is synthesized by complexes of Cellulose Synthase A (CESA) proteins (Mutwil et al., 2008; Handakumbura et al., 2013; Schwerdt et al., 2015). Lignin is an aromatic polymer made from covalent crosslinking of phenylpropanoid monomers. Lignin is characteristic of secondary cell walls and forms a barrier for breakdown of cellulose and other wall polysaccharides, including during biofuel production (Bonawitz and Chapple, 2010; Vanholme et al., 2010). Most lignin biosynthesis enzymes function similarly in eudicots as in grasses, though evidence of pathway differences is starting to emerge (Shen et al., 2012; Takeda et al., 2018). Phylogenetic analyses have revealed orthologs of lignin biosynthesis genes and CESAs across eudicots and monocots (Hazen et al., 2002; Penning et al., 2009; Popper et al., 2011; Carpita, 2012).

In contrast to cellulose and lignin, grass cell wall hemicelluloses differ from those of eudicots in composition and relative abundance (Liepman et al., 2010; Pauly et al., 2013). While in eudicots the major hemicellulose classes are xyloglucans, xylans, and mannans, in grasses, xylans and ß-(1,3;1,4) mixed-linkage glucan (MLG) are most abundant. Proteins of the grass-expanded glycosyl transferase (GT) 61 clade, including OsXAX1, OsXAT1, and OsXYXT1, add arabinose and more complex branches to grass xylans that are absent in most eudicots (Anders et al., 2012; Chiniquy et al., 2012; Zhong et al., 2018). Cellulose synthase-like (CSL) proteins, including OsCSLF6, OsCSLF8 and OsCSLH1, incorporate MLG into grass primary and secondary cell walls (Burton et al., 2006; Vega-Sanchez et al., 2012; Kim et al., 2015). Phylogenetic reconstruction suggests that the MLG-synthesizing CSLs emerged in grasses from expansion of the relatively conserved cellulose synthase-like gene families (Hazen et al., 2002; Popper et al., 2011). In addition, commelinid monocot lignin and arabinoxylan are esterified with the hydroxycinnamic acids, ferulic acid and *p*-coumaric acid. Several so-called “BAHD” acyl-CoA acyltransferases (ATs) from grasses hydroxycinnamoylate cell wall precursors (Withers et al., 2012; Bartley et al., 2013; Petrik et al., 2014; Karlen et al., 2016; De Souza et al., 2018). The cell wall BAHD clade includes 0 to 2 members in eudicots, but 10 to 20 members in commelinids (Karlen et al., 2016; De Souza et al., 2018). Detailed phenotypic analysis of the closest Arabidopsis ortholog failed to reveal a cell wall phenotype (Rautengarten et al., 2012), suggesting that the monocot enzymes may have evolved this function subsequent to the divergence of eudicots and commelinids, which is consistent with occurrence patterns of feruloylation of arabinoxylan (Harris and Trethewey, 2010). To facilitate communication, we refer to genes encoding the GT61, CSLF/H, and AT clades mentioned above as “grass-specific” relative to Arabidopsis.

Along with delineating cell wall synthesis enzymes, researchers have made significant progress in understanding regulation of secondary cell wall synthesis, especially for eudicots such as Arabidopsis and *Medicago*. More than 30 eudicot secondary cell wall regulators have been confirmed through detailed forward and reverse genetic analyses [reviewed in: (Zhong et al., 2007; Zhong et al., 2010; Wang and Dixon, 2012)]. A few NAC (NAM, ATAF1, 2 and CUC2) proteins are top-level activators (Mitsuda et al., 2007; Zhong et al., 2010; Zhou et al., 2014). For example, Arabidopsis NAC SECONDARY WALL THICKENINGS PROMOTING FACTOR1 (NST1), NST2, and Arabidopsis SECONDARY WALL-ASSOCIATED NAC PROTEIN1 (SND1, also known as NST3), function redundantly to activate overall secondary cell wall biosynthesis by enhancing expression of downstream transcription factors and cell wall biosynthesis genes (Appenzeller et al., 2004; Mitsuda et al., 2007; Wang and Dixon, 2012). Numerous R2R3 MYB family members function in secondary cell wall regulation (Zhao and Bartley, 2014). For example, AtMYB46 is a direct target of AtSND1 and can activate additional cell wall-associated transcription factors, *CESA*s, and lignin biosynthesis genes (Zhong et al., 2007; Ko et al., 2009; McCarthy et al., 2009; Zhong and Ye, 2014; Kim et al., 2014). Another example, AtMYB61, regulates plant resource allocation partly by activating cell wall synthesis genes and transcription factors (Newman et al., 2004; Romano et al., 2012). Recent large-scale promoter-transcription factor interaction experiments for Arabidopsis have expanded the likely cell wall-regulating transcription factor complement to over 200 proteins from multiple families and emphasized the feed-forward loop topology of the secondary cell wall biosynthesis regulatory network (Taylor-Teeples et al., 2015).

Fewer functional studies of cell wall regulation have been conducted in grasses [reviewed in: (Gray et al., 2012; Handakumbura and Hazen, 2012; Rao and Dixon, 2018)]. Our deepest understanding is arguably of a pair of negative regulators, ZmMYB31 and ZmMYB42, and their orthologs in rice, sorghum, and switchgrass, which repress secondary cell wall biosynthesis (Sonbol et al., 2009; Fornale et al., 2010; Rao et al., 2019) and bind to promoters of lignin biosynthesis gene *in vivo* (Agarwal et al., 2016). The Arabidopsis orthologs, AtMYB4 and AtMYB32, also repress lignin biosynthesis (Jin et al., 2000; Preston et al., 2004). Analysis of a battery of rice transgenics supports several orthologs of Arabidopsis secondary cell wall transcription factors as regulators of lignin biosynthesis (Hirano et al., 2013b). Similar results have been published recently with switchgrass (Rao et al., 2019). For example, OsMYB61a can bind to the promoters of and regulate secondary CESA expression (Hirano et al., 2013b; Huang et al., 2015). Likewise, AtSND1 orthologs in rice and other grasses (known as OsSWN1 in rice) activate secondary cell wall formation when expressed in Arabidopsis (Zhong et al., 2011) and when overexpressed in rice and switchgrass (Chai et al., 2015; Rao et al., 2019). From functional studies such as these, conservation of gene complement (Zhao and Bartley, 2014), and network analyses (Hirano et al., 2013b), secondary cell wall regulation appears to be conserved across angiosperms.

An outstanding gap that has not been systematically examined is the regulation of grass-specific cell wall genes. A recent analysis revealed that a trihelix family transcription factor, BdTHX1, binds to the promoter of Brachypodium *CSLF6* (Fan et al., 2018); however, the function of this gene has not been examined in eudicots. In general, grass-specific genes might be controlled either by novel or conserved regulators, or both.

To obtain an overview of grass cell wall regulation and distinguish between models of conservation and divergence in regulation of grass-specific genes, we turned to gene network analysis, which has been used successfully to decipher regulatory pathways and complex traits in many organisms (Movahedi et al., 2011; Mutwil et al., 2011; Yeung et al., 2011; Hirano et al., 2013a; Hirano et al., 2013b; Sarkar et al., 2014; Obertello et al., 2015; Taylor-Teeples et al., 2015). Rice gene coexpression networks include the Rice Oligonucleotide Array Database (ROAD), Oryza Express, RiceArrayNet, Rice GeneNet Engine, and RiceFREND (Lee et al., 2011; Cao et al., 2012; Ficklin and Feltus, 2013; Hirano et al., 2013a). Among these, ROAD and PlaNet permit download of the whole network, facilitating large-scale comparisons and cross-validation. Other so-called “functional gene networks” combine co-expression data with protein-protein interactions and other functional and physical association evidence. In particular, RiceNet (version 1, v1) of Lee et al. (2011) combines various co-expression and protein-protein interaction data from rice, Arabidopsis, *C. elegans*, human, and yeast to provide a Bayesian likelihood score of a functional association. Analysis of high-scoring genes from RiceNet showed that 13 of 14 previously unstudied network neighbors were capable of protein–protein interactions with “bait” genes and reverse genetics revealed that at least three of five genes function in the predicted process (Lee et al., 2011). This validation rate is much higher than typical for coexpression networks. For example, the Arabidopsis component of PlaNet gave a validation rate of ∼10% based on screening of T-DNA mutants for embryo lethality (Mutwil et al., 2011). This suggests that the approach of combining multiple types of data across multiple species provides a high-quality network with a reasonable ability to predict functional interactions. RiceNet v2 expands v1 by incorporating newer transcriptomics data and other updated genomic and molecular evidence (Lee et al., 2015). Similar co-expression and multi-data, Bayesian networks for Arabidopsis are also available (Obayashi et al., 2014; Lee et al., 2015).

Here, we combined publicly available, rice co-expression networks with a high-quality Bayesian network to create a novel, comprehensive, genome-scale network, the ice ombined mutual anked etwork (RCRN). Our goal was to study the regulation of grass cell wall biosynthesis relative to that of Arabidopsis. Our analysis suggests that orthologs of almost all Arabidopsis cell wall regulators are present in rice; however, some have different relative importance compared to those in Arabidopsis. Transient assays confirmed that four orthologs of Arabidopsis known cell wall transcription factor can activate cell wall biosynthesis genes. In addition, 6 out of 11 regulators that had not previously been examined for cell wall function control rice cell wall biosynthesis based on transient gene expression assays. Molecular genetics and direct binding assays show that OsMYB61a, a rice ortholog of the known cell wall regulator, AtMYB61, can bind to promoters of both CSL and AT grass-specific genes. This supports the model that grass-specific cell wall genes have been incorporated into regulatory cascades shared with eudicots.

## Methods

### Generation of the Rice and Arabidopsis Combined Ranked Networks

We constructed the RCRN to be a comprehensive, high-quality rice genome scale network based on three publically available networks, namely, ROAD, PlaNet and RiceNet v2. The goal of combining three networks is to expand the high quality network by recalibrating their association scores and covering more rice genes, which allows us to study grass-specific pathways. The three original rice networks, ROAD, PlaNet and RiceNet have three different score systems, Pearson Correlation Coefficient (PCC), Highest Reciprocal Rank (HRR) and Log Likelihood Score (LLS), respectively. For ROAD, we only included positive correlations with a score from 0.5 to 1 (Cao et al., 2012). PlaNet is a collection of different species networks and we only included the rice dataset into our study. PlatNet was built based on HRR and the score range is from 0 to 200 with increments of 1 (Mutwil et al., 2010; Mutwil et al., 2011). RiceNet v2 used log likelihood scores (LLS) to incorporate diverse proteomics, genomics and comparative genomics datasets likely related to rice biological process with scores ranging from 1 to 5 (Lee et al., 2010; Lee et al., 2011; Lee et al., 2015). To combine the three rice networks, we scaled different score systems using inverse mutual rank (MR) as follows: 1/MR = 1/sqrt (rank (A, B) × rank(B, A)) (Usadel et al., 2009). To apply the RiceNet scoring system to represent interactions between additional genes present in the other networks, we computed coefficients using a generalized linear (GLM) model in R based on 1,282 and 3,389 common edges among ROAD, PlaNet and RiceNet v2, respectively. This yielded the following equation:

(Equation I). $$\frac{1}{\mathrm{RiceNet}.v2\_\mathrm{MR}}=\frac{0.33}{\mathrm{ROAD}\_\mathrm{MR}}+\frac{0.025}{\mathrm{PlaNet}\_\mathrm{MR}}$$

We then rearranged equation I to calculate the RCRN, as follows:

(Equation II). $$\text{RCRN}=\frac{1}{\mathrm{RiceNet}.v2\_MR}+\frac{0.33}{\mathrm{ROAD}\_\mathrm{MR}}+\frac{0.025}{\mathrm{PlaNet}\_\mathrm{MR}}$$

To facilitate examination of the hub genes in rice and Arabidopsis cell wall networks, we also created the combined Arabidopsis network by integrating the functional network, AraNet v2, and co-expression network from ATTEDII. AraNet v2 used log likelihood score (LLS) to incorporate diverse proteomics, genomics and comparative genomics datasets likely related to rice biological process with scores ranging from 1 to 5 (Lee et al., 2010; Lee et al., 2015). For ATTED II, we only included positive correlations with the score from 0.5 to 1 (Aoki et al., 2015). A GLM yielded the Arabidopsis combined ranked network by calibrating ATTED II network edges based on AraNet using Equation III:

$$\text{ACRN}=\frac{1}{\mathrm{AraNet}.v2\_\mathrm{MR}}+\frac{0.27}{\mathrm{ATTED}\_\mathrm{MR}}$$

These networks are available for download at: https://doi.org/10.5061/dryad.zgmsbcc69

### Network Performance Assessment Based on Gene Ontology

We evaluated network quality based on Gene Ontology (GO) terms annotated by the Biofuel Feedstock Genomics Resources. In all, 40% of rice genes have been assigned the GO-biological process (BP) terms. As with assessment of RiceNet v2, we excluded 10 general GO-BP terms to avoid bias towards these common terms (Lee et al., 2015). We defined true positives as the number of edges with matched GO-BP terms with scores higher than a particular cutoff. True negatives are defined as the number of edges with unmatched GO-BP terms with scores lower than the cutoff. False positives are the number of edges unmatched GO-BP terms with scores higher than the cutoff. False negatives are defined as the number of edges with matched GO-BP terms with scores lower than the cutoff. For each network in the analysis, we applied 40 different cutoffs to generate the ROC curves by plotting true positive rate vs. false positive rate (Lee et al., 2004; Mcgary et al., 2007).

For the precision-recall analysis, precision was calculated as the proportion of true positive edges among all predictions at particular edge score cutoff. Recall represents the proportion of true positive edges relative to total true positives. Then, we defined the total number of edges with matched GO-BP terms within each whole (or trimmed) network as the Total True. At each network edge cut-off the fraction of True Positives (TP) is the number of edges with matched GO-BP terms over Total True Positives. The number of predictions (N) is defined as the number of edges within each network with a particular edge cut-off. Precision = TP/N. Recall = TP/Total True (Mcgary et al., 2007). As a control for this analysis, we built a random network by randomly assigning edges between a pair of genes within the rice genome.

### Network Comparisons

We constructed cell wall-only networks in Arabidopsis and rice by extracting interactions from the ACRN and RCRN without cutoffs. We used Inparanoid (Remm et al., 2001) to identify orthologs of Arabidopsis cell wall related genes in rice (http://inparanoid.sbc.su.se/cgi-bin/index.cgi) and phylogenetic reconstructions of the R2R3 MYB family (Zhao and Bartley, 2014). To compare the network connectivity between species, we isolated interactions between each rice transcription factor with its Arabidopsis ortholog and other cell wall-related genes and the total number of genes in RCRN and ACRN without edge score cut-off. If co-orthologs exist, we counted the union of interactions of both co-orthologs. Fisher’s exact test was used to determine the statistically different network connectivity for each set of (co-)orthologs.

### Transcription Factor Expression Patterns

Expression data for Arabidopsis cell wall-associated transcription factors were extracted from the Arabidopsis gene expression atlas (Schmid et al., 2005). For rice, data were extracted from the rice gene expression atlas (Wang et al., 2010). The gene expression heatmaps were plotted with the heat.map 2 function in R using default hierarchical clustering for row dendrograms.

### Construction of the Rice Cell Wall Network

To identify putative novel transcription factors controlling cell wall biosynthesis in rice, we constructed a 1-step network with 125 seed genes with the sum of inversed mutual rank score ≥0.03. This network includes 1,790 nodes and 215 of them are transcription factors. To better select candidates controlling rice cell wall biosynthesis, we excluded transcription factors with fewer than five edges with cell wall seed genes. In all, we predicted 96 transcription factors from 19 protein families as putative novel regulators of cell wall biosynthesis, as summarized in **Supplementary Table 4**.

### Characterization of *myb61a-1* Insertion Mutants

We characterized an insertion mutant line for *OsMYB61a*, *PFG_2D-10906*, called *myb61a-1*, which possesses the T-DNA insertion from *pGA2707* in *Oryza sativa japonica* cv. *Dongjin* (An et al., 2005; Jeong et al., 2006). The line *2D-10906-11* was found to be homozygous for the insertion and line *2D-10906-8* was identified and used as the negative segregant. Genotyping primers are listed into **Supplementary Table 6**.

We measured gene expression using the 5th leaf (numbered from the bottom) harvested from 2-month old plants, choosing morphologically and developmentally matched leaves for analysis, based on plant size, leaf length and expansion. RNA was extracted with a Zymo Quick RNA Extraction Kit. We used 1 µg RNA to synthesize cDNA with Promega MMLV reverse transcriptase kit. We ran quantitative PCR with BioRad SYBR Green Master Mix and BioRad CFX96 thermocycler. qPCR primers and locus IDs for genes measured in this study are listed in **Supplementary Table 7**. To analyze gene expression data, we first calculated the real-time primer efficiency with LinRegPCR (Ruijter et al., 2009). Gene expression data were normalized to two reference genes, *Cc55* and *Ubi5*, which show stable expression level during rice development (Jain, 2009). Student two-tailed t-tests were used to compare expression between wild-type and mutant plants. False positives were controlled using q-value to estimate the false discovery rate <0.05 (Lee et al., 2015).

### Cell Wall Assays

Five biological replicates of developmentally matched leaf and stem samples from 3-month old wild-type and *myb61a-1* plants were used for all cell wall assays. Alcohol insoluble residue (AIR) was prepared by boiling in 95% ethanol (1:4, w/v) for 30 min followed by washing with 70% ethanol and drying. Destarching to generate dsAIR, lignin *via* acetylbromide solubility, and cellulose content measurements were as previously described (Bartley et al., 2013). Mixed-linkage glucan (MLG) was measured by an enzyme-based kit (Megazyme, K-BGLU) with 5 mg of stem dsAIR as per the manufacturer’s directions. Cell wall-associated hydroxycinamic acids (e.g., FA and *p*CA) were examined in *myb61a-1* mutants and negative segregant plants. To release hydroxycinnamic acids from AIR, we treated 2 mg leaf AIR samples with 2 N NaOH for 24 h at 25 °C and analyzed the results with high performance liquid chromotography as described in Bartley et al. (2013). Student two-tailed t-tests were used to compare cell wall composition between wild-type and mutant plants.

### Transient Gene Expression Assay in Rice Protoplast

All transcription factors were cloned from *Kitaake* rice RNA into a pENTRY-D TOPO vector (Invitrogen) with primers summarized in **Supplementary Table 6**. *p2GW7* was used for overexpression (Karimi et al., 2007) in 2-week old, dark-grown *Kitaake* rice seedlings protoplast as previously described (Bart et al., 2006). Gene expression was measured through qPCR as described above with primers as listed in **Supplementary Table 7**.

A dexamethasone (DEX) inducible system was used to examine the downstream targets of OsMYB61a in rice protoplasts with and without the treatment of a protein inhibitor, cycloheximide (CHX). The *OsMYB61a:GR* sequence was cloned into the overexpression vector of *p2GW7* (Karimi et al., 2007) and transformed into protoplasts. DEX (10 µM) was used to induce translocation of OsMYB61a:GR from the cytoplasm to nucleus; control cells were treated with ethanol without DEX. Protein synthesis was blocked by treating protoplasts with 2 µm CHX 30 min prior and during DEX induction. Protoplast were cultured 8 h with DEX before RNA extraction. Four replicates were used in each assay.

### Yeast-One-Hybrid Assays

Full-length *OsMYB61a* coding sequence was cloned in-frame into the *GAL4* activation domain in the *pDEST22* vector (Invitrogen). Promoter fragments of (∼ > 1700 bp upstream of the transcription start site) of *OsCESA4*, *At3G62160*, *OsAT4*, *OsAT5*, *OsCSLF6*, and *OsCSLH1* were introduced into Gateway-compatible *pLUC* (*pLacZi* with replacement of *lacZ* with *gluc*) *via* LR recombination (Invitrogen), linearized with, and transformed into the *Saccharomyces cerevisiae* strain YM4271 (Deplancke et al., 2004; Deplancke et al., 2006) using 50% PEG-3350, 10× TE, and LiAc, as previously described (Walhout and Vidal, 2001). Transformations were plated in SD-U media at 30 °C for 2 days and colonies were grown in deep plates with 375 µl of SD-U liquid media in a shaking incubator. Before screening, baits were tested for self-activation of the *Renilla* luciferase reporter enzyme using native coelenterazine (nCTZ) substrate (Sigma-Aldrich). Cell culture (20 µl) were transferred to a 96-well flat bottom black plate (Greiner Bio-one) and luciferase activity was measured in a microplate reader (SpectraMax M5) upon addition of nCTZ substrate Mix (1X PBS, 5M NaCl, 1 mg/ml nCTZ solution). Luciferase activity in relative luciferase units (RLU) was normalized by optical density (600 nm). Non-self-active colonies were transformed with the prey, *OsMYB61a*, and empty vector control constructs and SD-TU medium was used for selection of bait-pray transformation. Data were calculated as average fold change relative to the empty expression vector for three biological and four technical replicates. A two-tailed t-test was used to identify statistically different means.

## Results

### Development of a High Coverage and High-Quality Rice Gene Network

Our goal was to utilize rice genome-scale networks to understand grass cell wall biosynthesis and regulation especially related to grass-specific aspects of the process. However, we found that the publicly available rice networks, ROAD, PlaNet and RiceNet v2, lacked some of the grass-specific cell wall genes available to use as “bait” genes, [including the 20 BAHD acyltransferase; 3 MLG biosynthesis genes, *OsCSLF6*, *OsCSLF8* and *OsCSLH1*; and 2 arabinoxylan modifying genes, *OsXAX1* and *OsXAT1* (grass-specific in **Supplementary Table 1**)]. The Bayesian functional network, RiceNet v2 lacks approximately one quarter of these genes (six, **Table 1**). Thus, this high-quality functional network may be incomplete with respect to grass-diverged cell wall synthesis. On the other hand, the two publicly available co-expression networks, ROAD and PlaNet, are only missing four and one of the grass cell wall genes, respectively. However, these rice co-expression networks have not been experimentally validated and may have lower predictive power, i.e., quality, compared with RiceNet v2.

**Table 1:** Summary of network features.

Network (Citation)	Network Scoring System	Included grass-specific cell wall genes^a^	Total nodes	Total edges	R^2^ of the power law^b^
ROAD (Cao et al., 2012)	Pearson correlation coefficient (PCC)	21	28,626	8,520,163	0.76
PlaNet (Mutwil et al., 2011)	Highest reciprocal rank (HRR)	24	30,428	3,310,397	0.54
RiceNetv2 (Lee et al., 2015)	Bayesian log likelihood probability	19	25,765	1,775,000	0.88
RCRN (this study)	Sum of inverse rank (SIR)	24	36,419	13,185,506	0.74
ACRN (this study)	Sum of inverse rank (SIR)	NA^c^	24,648	5,319,766	0.69

a Included grass-specific cell wall genes indicates the number out of 25 genes in this category as described in the text and (listed in **Supplementary Table 1**).

b The power law distribution is P(k) ∼ k^γ^, which represents the probability of a node with k edges with γ as a constant for a given biological network.

c Not applicable.

To overcome the potential depth and quality limitations of existing networks, we developed a genome-scale integrated network suitable for mining grass-diverged traits. Our heuristic strategy was to use a generalized linear model (GLM) to recalibrate the edge scores between genes within ROAD and PlaNet to the scoring system of RiceNet v2. To scale the different scores to a similar range, we first calculated the inverse mutual rank for each network based on their original scores. Inverting the ranking makes greater scores reflect greater confidence. For ROAD, we used only positive correlations; whereas, positive scores for RiceNet and PlaNet include both positive and negative co-expression correlations. The result was the Rice Combined mutual Ranked Network (RCRN) (see Equations I and II in *Methods*).

**Figure 1:**
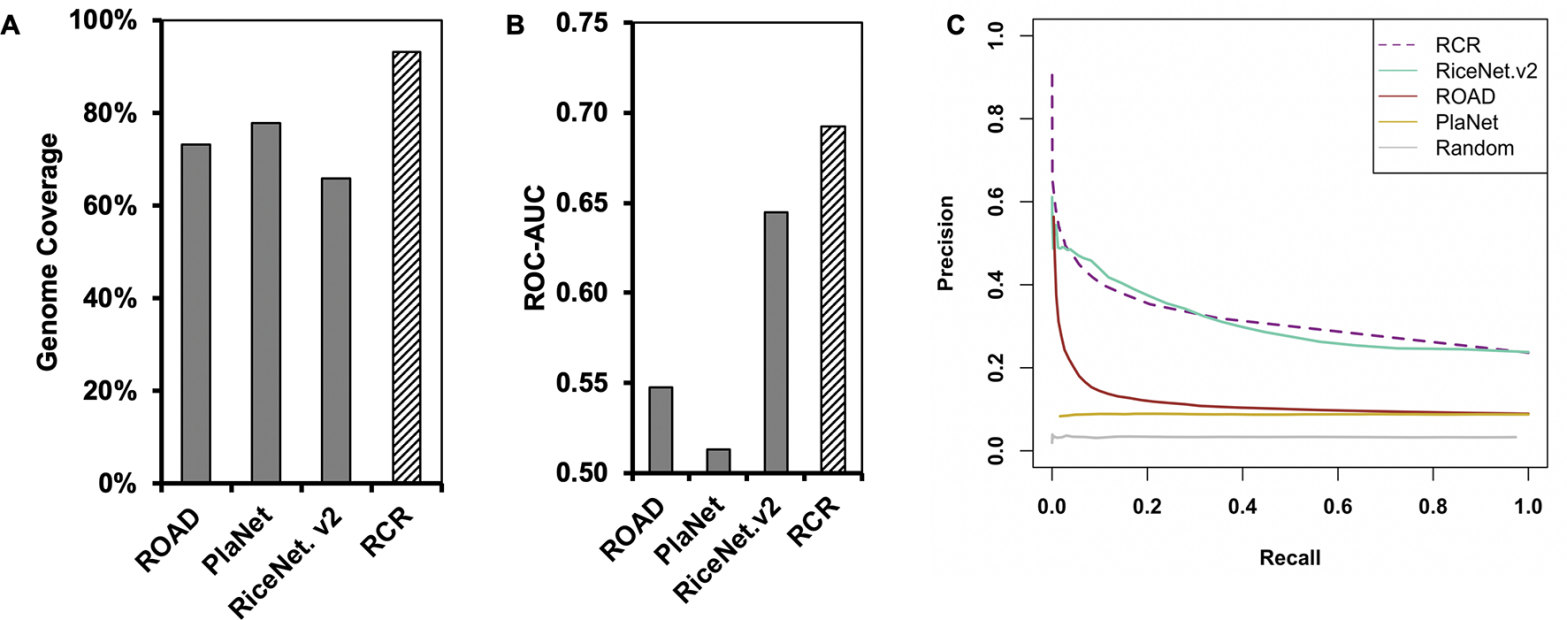
The Rice Combined inverse mutual Ranked (RCR) network (hashed bar) shows greater genome coverage and higher quality compared to published networks (solid bars). **(A)** Genome coverage of the networks. **(B)** The Receiver Operating Characteristic Area Under the Curve (ROC-AUC) indicates that the RCRN has better predictive power compared to the original networks. The curve was generated based on matching of GO-Biological Process (BP) annotation between network nodes at different edge-value cut-offs. **(C)** Precision-recall analysis supports that the RCRN has similar or better true positive prediction performance than the original networks. ROAD represents Rice Oligonucleotide Array Database (Cao et al., 2012); PlaNet represents the rice genome-scale network from the PlaNet database (Mutwil et al., 2010); RiceNet. v2 represents the rice Bayesian network, version 2 (Lee et al., 2015). Random is a simulated network with randomized edges.

Compared to the original rice networks, the RCRN shows the highest genome coverage and maintains a scale-free topology. The RCRN covers 93% of rice genes (**Figure 1A**, **Table 1**) and misses only one (4%) of our list of grass-specific cell wall genes. This suggests that the RCRN is effective for study of specialized genes or traits of rice and other grasses. Moreover, we analyzed the topology of the networks by calculating fitness to the power law distribution, since biological networks have been found to be scale-free, with a few nodes having a very large number of edges (Barabási and Oltvai, 2004; Siegal et al., 2007; Baxter et al., 2015). All the rice networks fit the power law, though PlaNet fits least well (**Table 1**).

Besides improved genome coverage, the RCRN shows the highest predictive power compared to the three previous networks based on Gene Ontology (GO)-based evaluation. As genes involved in the same pathway tend to be co-expressed and co-regulated (Ashburner et al., 2000; Chang et al., 2013), we evaluated network quality based on Biological Process (BP) GO terms from the Biofuel Feedstock Genomics Resource (Childs et al., 2012). Forty percent of rice genes have been assigned GO-BP terms. We excluded 10 common GO-BP terms to avoid bias from these high-level, generic terms (Lee et al., 2015). A Receiver-Operating Characteristic (ROC) curve measures the predictive power of each network at a series of edge score cut-offs. The ROC indicates the ratio of likely true positives with matched GO-BP terms compared the likely false positives with unmatched GO-BP terms. The area under the ROC curve (AUC) is higher for the RCRN (AUC = 0.69) than for the other networks (**Figure 1B** and **Supplementary Figure 1**). Precision-recall analysis, which focuses only on true positive predictions at different edge scores, also suggests that the RCRN exhibits a greater proportion of positive edges compared to the co-expression networks and a similar proportion to that of RiceNet v2 (**Figure 1C**).

### Comparison of the Rice and Arabidopsis Cell Wall Regulatory Networks

To compare rice and Arabidopsis cell wall regulation, we first tested recall of known cell wall-related interactions in the RCRN by extracting edges between the cell wall “bait” (target) genes in three categories, 1) functionally characterized rice cell wall biosynthesis gene families including those of phenylpropanoid pathway genes, cellulose synthases, “Mitchell-Clade” BAHD acyltransferases, and xylan biosynthesis genes; 2) known grass cell wall-associated transcription factors; and 3) putative orthologs of known Arabidopsis cell wall-associated transcription factors (**Supplementary Table 1**). These 125 cell wall genes are highly interconnected in the RCRN, with their graph possessing 1177 edges when considered without edge-score cut-offs (**Supplementary Figure 2**). This recalls 92% (97 out of 105) of rice orthologs of known transcription factor-cell wall biosynthesis gene associations (**Supplementary Table 2**).

**Figure 2:**
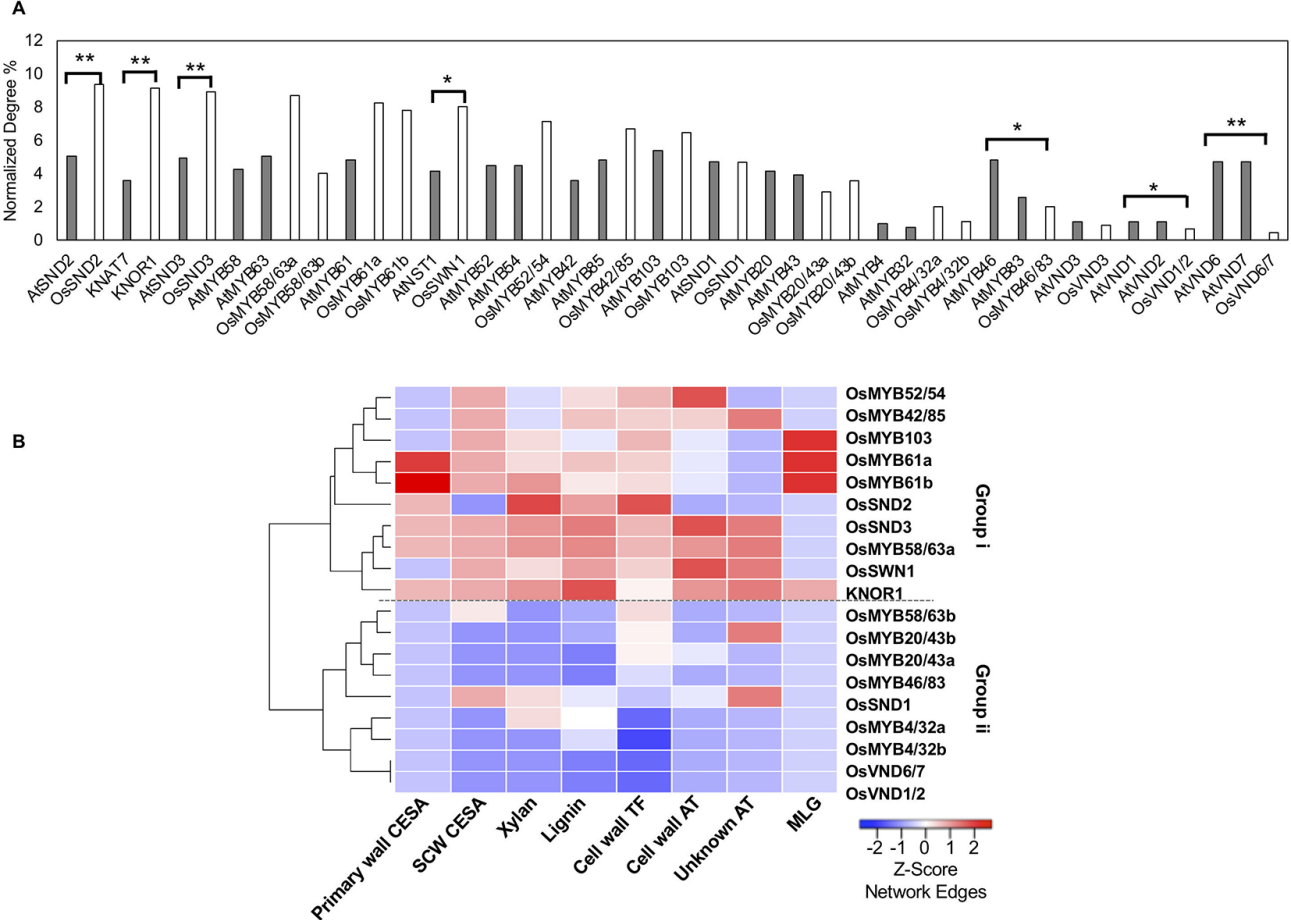
Rice (co-)orthologs of Arabidopsis cell wall transcription factors possess varied numbers of edges in the rice cell wall network. **(A)** Some transcription factor (co-) orthologs have a significantly different normalized number of interactions with cell wall genes within the rice (solid bars) and the Arabidopsis (hatched bars) networks. *indicates Fisher test p value < 0.05 ; **indicates Fisher test p value < 0.01. **(B)** Rice transcription factors cluster into two groups (i and ii) depending on the number of edges with different cell wall gene classes. The heatmap displays z-score normalization of the number of interactions with transcription factors for each group of cell wall-related genes, as extracted from the RCRN without edge cutoffs. See **Supplementary Table 1** for a summary of gene abbreviation explanations and locus IDs.

We then created the Arabidopsis Combined mutual Rank Network (ACRN) and extracted a similarly constructed cell wall network including genetically verified regulators, lignin, and cellulose synthesis genes. Like the RCRN, the ACRN combines the Arabidopsis Bayesian functional network, AraNet v2, and the co-expression network, ATTED II, through a GLM. Based on the number of edges with cell wall-related genes in the ACRN, many regulators are highly connected hubs, including AtSND1, AtSND2, AtSND3, AtNST1, AtVND6, and AtVND7, and their targets, including, AtMYB103, AtMYB63, and AtMYB46, among others (**Figure 2A**, **Supplementary Figures 2A**, **3**). That many genetically verified Arabidopsis cell wall regulators possess relatively high numbers of edges in the ACRN is consistent with the observation that “important” regulators are well connected within gene networks (Sorrells and Johnson, 2015). Additionally, many of these hub regulators are highly expressed in Arabidopsis stems compared to relatively less connected regulators in gene expression atlas data (**Supplementary Figure 4**).

We conducted a similar examination of rice orthologs of eudicot cell wall transcription factors in the cell wall network derived from the RCRN (**Supplementary Figure 2B**) and compared the results with those for Arabidopsis. To compare across species, we calculated the union of cell wall edges for co-orthologs (e.g., one gene in Arabidopsis vs. two genes in rice). The rice network has a more varied degree distribution, but still most orthologous gene sets possess similar numbers of edges between the rice and Arabidopsis networks (**Figure 2A**, **Supplementary Table 3**). For example, co-orthologs of AtMYB58 and AtMYB63, OsMYB58/63a and OsMYB58/63b are still highly connected, hub regulators. On the other hand, OsVND6/7, OsVND1/2 and OsMYB46/83 show significantly lower relative degree compared to their co-orthologs in Arabidopsis and relatively low gene expression (**Supplementary Figure 5**). In contrast, the rice ortholog of KNAT7 (named KNOTTED 1 of ice, KNOR1), OsSND2, OsSND3, and OsSWN1 possess significantly R more cell wall edges than their orthologs do in Arabidopsis (**Figure 2A**) and are among the more highly expressed putative rice cell wall transcription factors (Supplementary Figure 5). Beyond connections with just cell wall-related genes, we also investigated the connectivity using the total number of edges in the RCRN versus the ACRN and observed that most transcription factors show conserved connectivity, but a few are shifted (**Supplementary Figure 6**).

We further categorized the networks of rice cell wall-related genes based on the components that they synthesize (**Figure 2B**). Group i members have high degree with lignin and xylan biosynthesis genes and both secondary and primary cell wall CESAs. Group ii members show relatively lower degree with the classes of cell wall genes considered. The network connectivity and gene expression analysis suggest cases of both conserved and shifted importance in the cell wall biosynthesis regulatory networks between Arabidopsis and rice.

### Identification of Additional Cell Wall-Associated Transcription Factors

To systematically identify transcription factors that may control rice cell wall biosynthesis, we examined the higher confidence edges of the Rice Cell Wall Network. This network extends from the 125 cell wall “bait genes” to include nodes from the RCRN with a sum of inverse rank (SIR) edge-score ≥0.03, i.e., the top 30 mutual rank interactions for each bait for a total of 1,790 non-bait nodes and 3,139 edges (**Supplementary Figure 7**). Of these, 215 are annotated as transcription factors and 96 connect with at least five cell wall bait genes. These 96 highly connected transcription factors are from 19 protein families, including multiple members of the MYB, NAC, TALE, AP2/ERF, HD-ZIP, bHLH, WRKY, DBB, C2H2, GATA, ARF, and MICK families along with seven others (**Supplementary Tables 4** and **5**). Twenty-one of the 96 high-degree transcription factors in the RCRN overlap with the novel transcription factors that are highly co-expressed (mutual rank >55) in a rice secondary cell wall network (Hirano et al., 2013a) (**Supplementary Table 5**). Furthermore, 79 have orthologs in Arabidopsis and 58 of those (73%) are part of the cell wall network in the ACRN, 16 (20%) of which have high degree with Arabidopsis secondary cell wall genes (**Supplementary Table 5**), consistent with conservation of cell wall association.

Based on their connection patterns with cell wall biosynthesis genes, the 96 putative uncharacterized wall-associated transcription factors can be divided into three groups (**Supplementary Figure 8**). Group i member share edges with most categories of cell wall genes, except primary cell wall CESAs and MLG biosynthesis genes. Group ii members are relatively less connected; however, a few show specific connections with primary cell wall CESAs and MLG biosynthesis genes. Group iii members connect mostly with cell wall transcription factors (**Supplementary Figure 8**).

### Recruitment of Grass-Specific Cell Wall Genes to a Conserved Regulatory Network

We next conducted functional analysis to validate the RCRN and explore regulation of grass-specific cell wall genes. OsMYB61a is one of two grass co-orthologs of AtMYB61, an activator of cell wall synthesis and other carbon-sink physiology (Romano et al., 2012). The RCRN suggests that OsMYB61a regulates CESA and lignin biosynthesis genes, as previously observed (Hirano et al., 2013b; Huang et al., 2015), and further, that OsMYB61a may control grass-specific cell wall genes (**Figure 2B**). To test this, we characterized a mutant line, *myb61a-1*, which has a T-DNA insertion in the third exon (**Supplementary Figure 9A**). Quantitative reverse transcription PCR (qRT-PCR) indicated that expression of *OsMYB61a* decreases at least five-fold in mature leaves of the mutant compared to those of negative segregant, wild-type plants (**Supplementary Figure 9B**).

**Figure 3:**
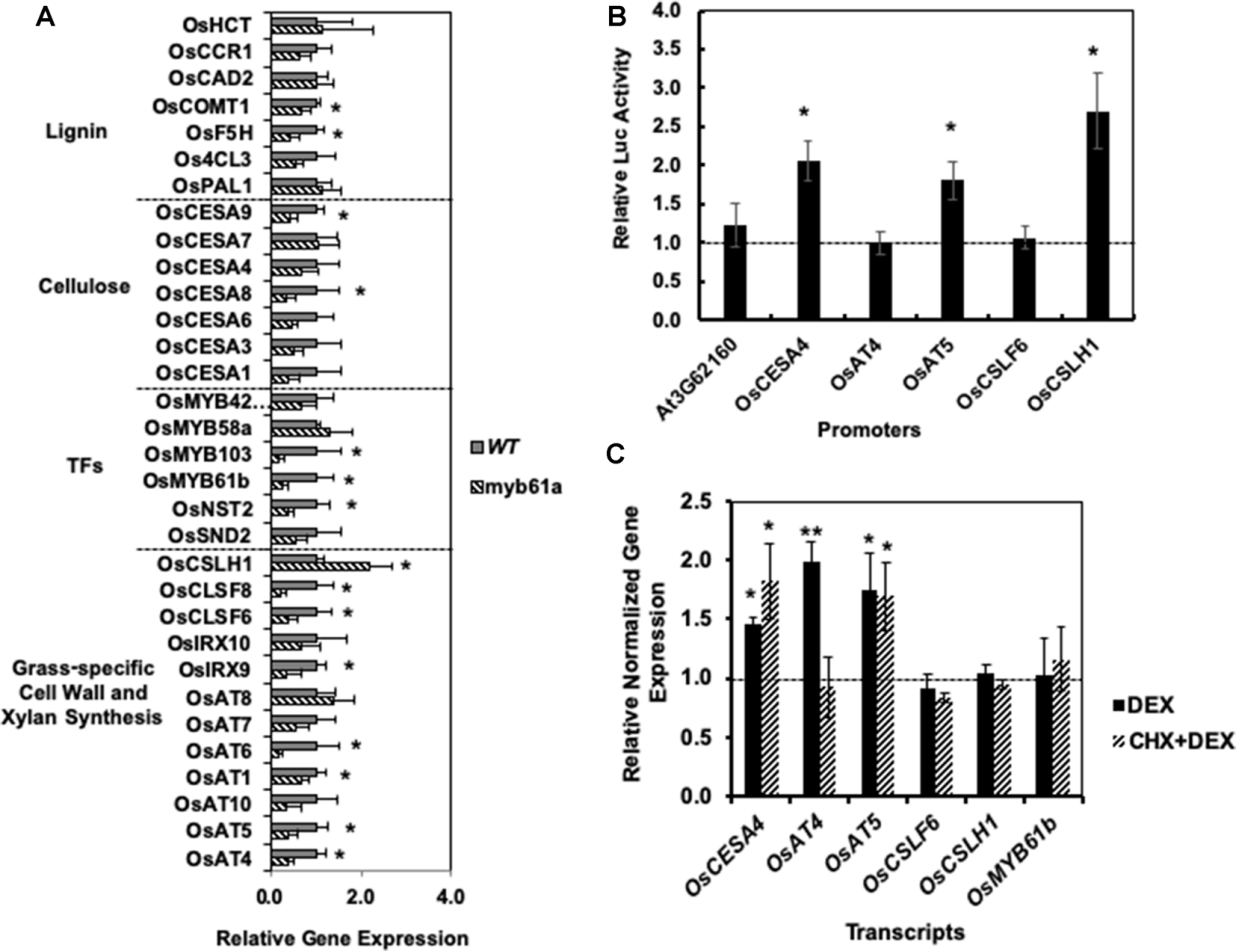
Reverse genetics and *cell-based* assays suggest that OsMYB61regulates numerous cell wall genes and directly bind to and activates promoters of grass cell wall-specific genes. **(A)** In *myb61a-1* mutant (hatched), many cell wall-related gene expressions have altered expression relative to the wild type (solid). Shown is average gene expression measured by qRT-PCR with two reference genes from five biological replicates of leaf samples from two-month old rice plants. Error bars represent the standard deviation. * indicates a false discovery rate (FDR) q-value < 0.05. **(B)** Yeast one-hybrid assay results in terms of the average fold change of luciferase (Luc) reporter activity due to expression of OsMYB61a fused to the GAL4 activation domain relative to an empty vector construct with the activation domain alone. Error bars are twice the standard deviation of three biological replicates. **(C)** Average normalized relative gene expression measured *via* qRT-PCR for rice protoplast transformed with OsMYB61a-GR and then induced with dexamethasone (DEX) or treated with translation inhibitor cycloheximide (CHX) prior to and during DEX induction. Expression is relative to *Ubq5* and *CC55* reference genes and normalized to expression in cells not treated with DEX. Error bars represent the standard deviation of four biological replicates. *indicates a difference from 1.0 at p < 0.05, and **indicates p < 0.01 *via* two-tailed t-test.

Guided by potential regulatory interactions inferred from edges in the RCRN, we tested 32 cell wall-related genes for alterations in expression in *myb61a-1* mutant plants with qRT-PCR (**Figure 3A**). Fourteen genes show a change in gene expression with an average fold-change of 3 ( ± 1)-fold (q-value <0.05; **Figure 3A**). Expression of lignin biosynthesis genes, *OsCOMT1* and *OsF5H1*, and the secondary cell wall cellulose biosynthesis gene, *OsCESA9*, are modestly, but significantly reduced relative to wild-type plants. In addition, expression of all grass-specific cell wall genes connected with *OsMYB61a* in the RCRN, except for *OsCSLH1*, was significantly reduced in *myb61a-1* compared to in the wild-type (**Figure 3A**). Surprisingly, *OsCSLH1* showed increased expression in *myb61a-1* (2.2-fold, q = 0.04). Though lacking a connection with OsMYB61a in the RCR, two additional BAHD AT-encoding genes, *OsAT1* and *OsAT6*, also showed reduced expression in *myb61a* (**Figure 3A**), though *IRX10*, *OsAT7*, *OsAT8*, and *OsAT10* did not.

To examine whether OsMYB61a controls a regulatory cascade in rice, we measured the expression of six orthologs of Arabidopsis secondary cell wall-associated transcription factors that both share an edge with OsMYB61a and other cell wall synthesis genes in the RCRN and display relatively high expression in rice vegetative development (**Supplementary Figure 5**). *OsMYB61b*, *OsNST2*, and *OsMYB103* all show reduced expression in *myb61a-1* relative to wild type (**Figure 3A**), with *OsMYB103* showing the greatest reduction in expression of any gene assayed at sixfold, consistent with being downstream of OsMYB61a in the rice cell wall transcriptional network.

**Figure 4:**
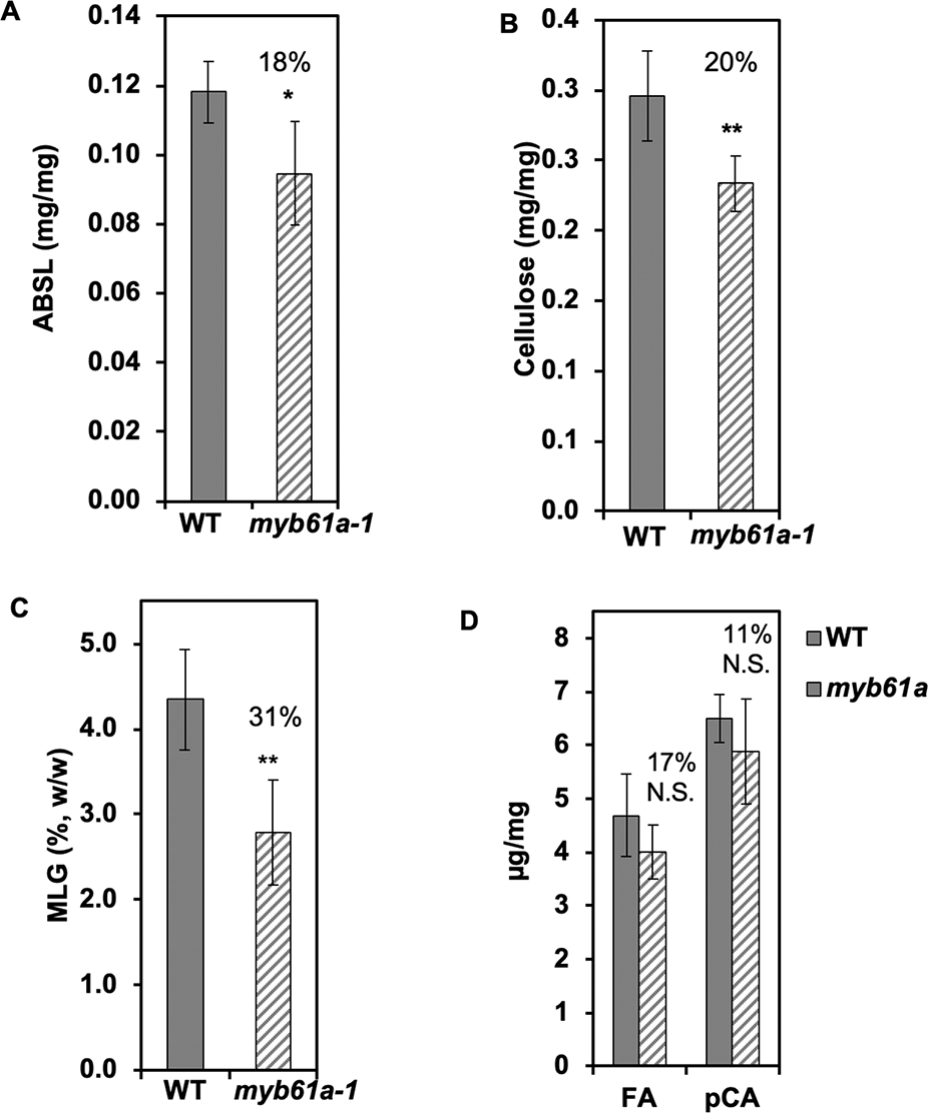
Secondary cell wall components decrease in *myb61a-1* (hatched) relative to the wild type (solid). **(A)** Average acetylbromide soluble lignin (ABSL) lignin of leaves. **(B)** Average cellulose content of leaves measured by the anthrone assay. **(C)** Average mixed-linkage glucan content of stems *via* the lichenase assay. **(D)** Hydroxycinnamates, ferulic acid (FA) and *p*-coumaric acid, of leaves. All measurements are from developmentally matched three-month old plant samples and expressed in terms of mass of alcohol insoluble cell wall residue (AIR), except for the cellulose measurement, in which case the AIR was destarched. Numbers indicate the percent reduction relative to the wild type. Five biological replicates were used. Error bars represent the standard deviation. N.S. indicates not significant, *indicates p < 0.05, and **indicates p < 0.01 *via* Student’s two-tailed t-test.

As expected from the reduction of expression of several cell wall synthesis genes, we found that *myb61a-1* mutant rice leaves and stems exhibit various cell wall phenotypes. Relative to the wild type, acetyl bromide soluble lignin (ABSL) and cellulose content of *myb61a-1* were reduced by 18% (p < 0.05) and 20% (p < 0.01), respectively (**Figure 4**). Furthermore, a lichenase assay showed that the grass-specific polymer, MLG, is reduced by 31% (p < 0.01) in mature *myb61a-1* stems (**Figure 4**). Finally, saponification of cell wall alcohol-insoluble residue (AIR) of leaf samples revealed a trend in reduction of FA and *p*CA of 17% and 11%, respectively (**Figure 4**), though these changes are not statistically significant (p = 0.2 and 0.3, respectively). Consistent with a defect in cell wall structural strength, *myb61a-1* plants also show a dwarf phenotype relative to the wild type (36% decrease, p < 10^5^), with each internode of *myb61a-1* being smaller than those of the wild type (**Supplementary Figures 9C, D**).

Next, we assessed whether OsMYB61a can directly bind promoters of grass cell wall biosynthesis genes in two assays. Yeast one-hybrid assays show that OsMYB61a directly binds to the ∼1.7-kb promoters of *OsCESA4*, *OsAT5*, and *OsCSLH1* (**Figure 3B**). As a negative control for this experiment, we tested the interaction between OsMYB61a and the promoter of *AT3G62160*, the Arabidopsis homolog of the rice “Mitchell clade” BAHD-acyltransferases, a knockout of which lacks a cell wall hydroxycinnamate phenotype (Rautengarten et al., 2012). We also analyzed the ability of OsMYB61a to directly alter transcription of grass-specific genes in rice seedling leaf-derived protoplasts when regulated by dexamethasone (DEX) with and without treatment with the protein biosynthesis inhibitor, cycloheximide (CHX). We observed that upon DEX-induction, an OsMYB61a-glucocorticoid receptor ligand-binding domain (GR) fusion protein activated expression of *OsCESA4*, *OsAT4*, and *OsAT5*. However, only *OsCESA4* and *OsAT5* were still induced after treatment with CHX, suggesting that OsMYB61a binds directly to these promoters. In contrast *OsAT4* expression activation may rely on interactions with another transcription factor client induced by OsMYB61a (**Figure 3C**). Thus, OsMYB61a is able to directly regulate expression of some grass-specific cell wall genes that eudicots lack.

### Functional Validation of Orthologs of Arabidopsis Cell Wall Transcription Factors

To accelerate functional exploration of the RCRN, we tested the ability of four orthologs of known cell wall regulators to alter cell wall-related gene expression in rice seedling-derived protoplasts. Transient transcription factor overexpression was driven by the cauliflower mosaic virus *35S* promoter, which is moderately strong in grass cells (Terada and Shimamoto, 1990).

To test the transient protoplast assay sensitivity and accuracy, we overexpressed *OsMYB61a* and were able to recapitulate many gene expression changes expected from the whole plant studies. We observed that four of nine genes that were decreased in leaves of *myb61a-1* knockout plants increased (1.5- to 2-fold, P < 0.05, **Figure 3A**) in protoplasts over expressing *OsMYB61a*, including, *OsF5H1*, *OsCESA9*, *OsCSLF6* and *OsAT4* (**Table 2**). From this, we conclude that this assay may be less sensitive than whole plant genetic manipulation, but nonetheless, the results support the conclusion that OsMYB61a activates grass-specific cell wall genes.

**Table 2:** Expression of cell wall biosynthesis genes in rice protoplasts overexpressing rice (co-)orthologs of Arabidopsis secondary cell wall transcription factors.

	LOC_Os Name	01g18240 OsMYB61a	05g04820 OsMYB61b	02g46780 OsMYB58/63a	01g48130 OsSND2
Transcription Factors		*190 ± 30^a^*	*320 ± 10^b^*	*590 ± 20^b^*	*270 ± 10^b^*
Lignin	*Os4CL3*	1.0 ± 0.2	*1.4 ± 0.2^a^*	*4.7 ± 0.3^a^*	ND^c^
	*OsCCR1*	1.2 ± 0.2	1.3 ± 0.2	1.2 ± 0.2	1.0 ± 0.1
	*OsCOMT1*	1.3 ± 0.3	*1.6 ± 0.1^a^*	*5.1 ± 0.7^a^*	0.8 ± 0.1
	*OsF5H1*	*1.6 ± 0.2^a^*	*1.6 ± 0.2^a^*	*1.8 ± 0.1^a^*	0.9 ± 0.1
	*OsCAD2*	ND^c^	ND^c^	*2.5 ± 0.2^a^*	1.3 ± 0.2
Secondary wall CESA	*OsCESA4*	ND^c^	ND^c^	0.9 ± 0.1	0.4 ± 0.3
	*OsCESA9*	*1.9 ± 0.2^a^*	*2.1 ± 0.3^a^*	1.0 ± 0.3	*0.3 ± 0.1^a^*
Mixed-linkage glucan	*OsCSLF6*	*1.6 ± 0.1^a^*	1.3 ± 0.1	ND^c^	ND^c^
	*OsCSLH1*	1.1 ± 0.1	1.1 ± 0.2	ND^c^	ND^c^
Hydroxycinnamic acid	*OsAT4*	*1.8 ± 0.1*^a^	*1.7 ± 0.1*^a^	1.8 ± 0.2^a^	0.3 ± 0.2^a^
	*OsAT5*	1.3 ± 0.2	2.0 ± 0.3^a^	1.1 ± 0.1	*0.3 ± 0.1*^a^

Data represent average fold change and standard deviation of the normalized expression (based on reference genes, Ubi5 and CC55) in three biological replicates upon expression of the regulators under control of the 35S promoter relative to empty vector controls. Data are from a single representative experiment. All experiments were repeated independently two to three times and bold and italic font demarcates repeatable significant differences.

a Two-tailed t-test p-value < 0.05.

b Two-tailed t-test p-value < 0.01.

c ND indicates the interactions were not determined in this assay, which examined interactions based on RCR network predictions.

**Figure 5:**
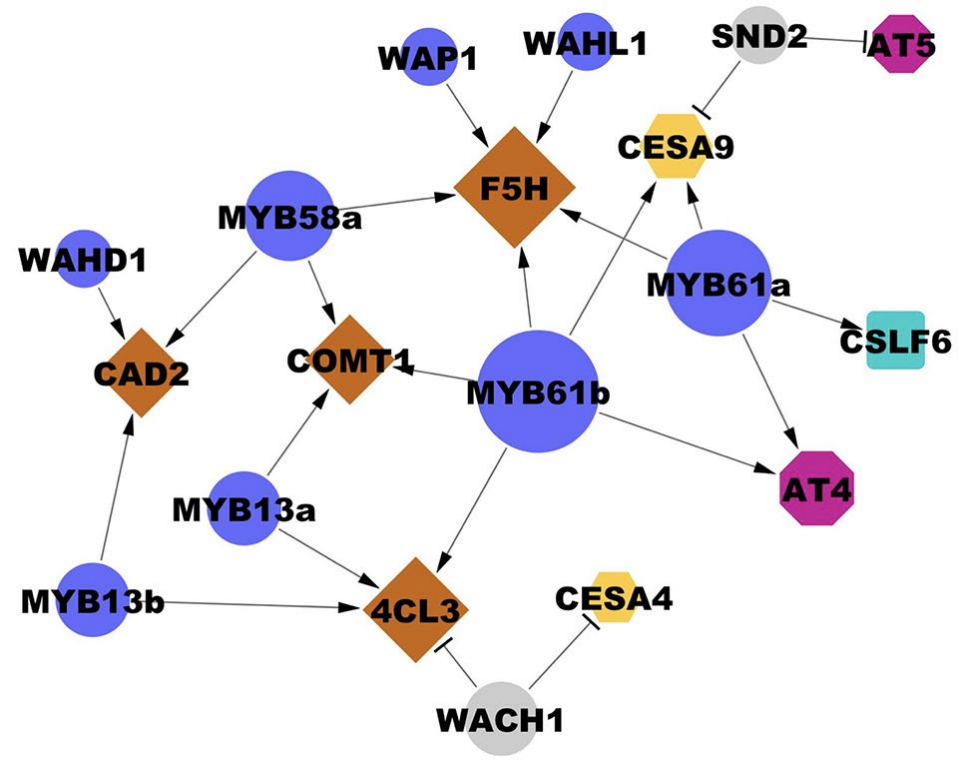
Interactions between transcription factors and cell wall biosynthesis genes validated in the transient assay. Edges ending in a triangle (i.e., arrow head) indicate activation; edges ending in a bar indicate repression. Gene node size is proportional to degree (number of regulatory connections). Blue and grey circles represent cell wall-associated activators and repressors, respectively. Brown diamonds represent lignin biosynthesis genes. Yellow hexagons represent CESAs. Magenta octagons represent cell wall-associated acyltransferases (AT). The blue square represents a mixed-linkage glucan biosynthesis gene. Locus IDs of transcription factors are listed in **Table 2** and **3** and locus IDs of all genes are in **Supplementary Table 1**. Three biological replicates were used in each experiment and each result was observed in at least two independently replicated experiments.

Next, we examined the effect on cell wall gene expression of overexpression of orthologs of three other characterized Arabidopsis genes, *OsMYB61b*, *OsMYB58/63a* and *OsSND2*, which may also act as hub regulators of rice cell wall synthesis based on network connectivity (**Figure 2**). We found that OsMYB61b, a paralog of OsMYB61a, also activates lignin and cellulose biosynthesis gene expression (Koshiba et al., 2017), and the grass-specific cell wall genes, *OsAT4* and *OsAT5* (**Table 2**). A co-ortholog of the Arabidopsis lignin biosynthesis transcriptional activator (Zhou et al., 2009), OsMYB58/63a activates four out of five tested lignin biosynthesis genes in protoplasts (**Table 2**, **Figure 5**), consistent with the rice protein having a conserved function with AtMYB58/63. Unexpectedly, OsSND2 may repress cell wall synthesis gene expression in rice, as transient overexpression of *OsSND2* reduced expression of *OsAT5* and *OsCESA9* by approximately 3-fold (**Table 2**, **Figure 5**). The literature reveals some ambiguity in the Arabidopsis ortholog’s role as a positive or negative regulator (Zhong et al., 2008; Hussey et al., 2011).

### Functional Validation of Novel Putative Rice Cell Wall Regulators

To extend our understanding of secondary cell wall regulation, we selected eleven unstudied, putative cell wall transcription factors from the 96 high degree rice transcription factors in the RCRN (**Figure 5**, **Supplementary Figure 8**). For each transcription factor, we tested its ability to alter expression of cell wall genes with edges in the RCRN. Transient overexpression in rice protoplast showed statistically significant and repeatable alterations in expression of cell wall genes for 55% (6 out of 11) of the uncharacterized transcription factors consistent with these proteins regulating cell wall biosynthesis (**Table 3**, **Figure 5**).

**Table 3:** Expression of cell wall biosynthesis genes in rice protoplasts overexpressing putative cell wall transcription factors. Data represent average fold change and standard deviation of three biological replicates of normalized expression (based on expression of references genes, *Ubi5* and *CC55*) upon expression of the regulators under control of the *35S* promoter relative to empty vector controls. Data are from a single representative experiment. All experiments were repeated independently two to three times and bold and italic font demarcates repeatable significant differences.

	LOC_Os Gene/Family	02g41510 MYB13a	04g43680 MYB13b	03g08470 WAP1	01g11910 WAHL1	12g43950 WAHD1	04g08060 WACH1	10g39030 bHLH	01g39330 bHLH	06g43860 KNOX	06g46270 NAC	07g48550 NAC
Transcription factors		*162 ± 12^a^*	*209 ± 31^b^*	*95 ± 16^b^*	*365 ± 34^b^*	*164 ± 12^b^*	*736 ± 49^b^*	*164 ± 20^b^*	*216 ± 19^b^*	*289 ± 37^b^*	*318 ± 19^a^*	*371 ± 26^b^*
Lignin	*Os4CL3*	*1.4 ± 0.1^a^*	*1.7 ± 0.1*^a^	1.1 ± 0.1	1.3 ± 0.1	1.0 ± 0.1	*0.4 ± 0.1*^a^	1.2 ± 0.1	1.3 ± 0.2	1.0 ± 0.1	1.2 ± 0.1	1.0 ± 0.1
	*OsCCR1*	1.6 ± 0.2	1.4 ± 0.2	1.0 ± 0.4	1.2 ± 0.1^a^	1.0 ± 0.1	1.0 ± 0.2	1.2 ± 0.1	1.1 ± 0.1	1.1 ± 0.1	1.2 ± 0.1	1.0 ± 0.1
	*OsCOMT1*	*1.6 ± 0.1^a^*	1.1 ± 0.1	1.0 ± 0.2	1.1 ± 0.1	0.9 ± 0.1	0.6 ± 0.1^a^	0.9 ± 0.1	0.9 ± 0.2	1.0 ± 0.1	1.0 ± 0.1	1.1 ± 0.1
	*OsF5H1*	2.4 ± 0.5^a^	1.3 ± 0.1	*2.0 ± 0.2^a^*	*1.4 ± 0.1^b^*	ND^c^	2.3 ± 0.3^a^	0.8 ± 0.1	1.2 ± 0.1^a^	1.0 ± 0.1	1.2 ± 0.2	1.1 ± 0.1
	*OsCAD2*	0.8 ± 0.1	*1.4 ± 0.1^a^*	0.9 ± 0.1	ND^c^	*1.8 ± 0.2^a^*	1.0 ± 0.2	0.8 ± 0.1	ND^c^	ND^c^	ND^c^	ND^c^
Secondary wall	*OsCESA4*	ND^c^	ND^c^	1.2 ± 0.2	1.3 ± 0.1	1.5 ± 0.3	*0.2 ± 0.1^a^*	0.9 ± 0.2	1.3 ± 0.1	0.9 ± 0.1	1.0 ± 0.3	1.0 ± 0.1
CESA	*OsCESA9*	ND^c^	ND^c^	1.2 ± 0.3	ND^c^	1.0 ± 0.1	1.6 ± 0.4	1.2 ± 0.01	1.1 ± 0.1	0.6 ± 0.2	1.6 ± 0.2	1.1 ± 0.1
MLG	*OsCSLF6*	ND^c^	ND^c^	1.1 ± 0.2	ND^c^	1.0 ± 0.1	0.9 ± 0.4	ND^c^	ND^c^	ND^c^	ND^c^	ND^c^
	*OsCSLH1*	ND^c^	ND^c^	1.4 ± 0.2	ND^c^	1.1 ± 0.1	1.1 ± 0.3	ND^c^	ND^c^	ND^c^	ND^c^	ND^c^
Hydroxycinnamic acid	*OsAT4*	1.0 ± 0.1	1.0 ± 0.1	1.0 ± 0.1	1.2 ± 0.1	1.2 ± 0.4	1.8 ± 0.1^a^	0.8 ± 0.2	1.0 ± 0.2	ND^c^	1.2 ± 0.3	0.7 ± 0.3
	*OsAT5*	1.0 ± 0.2	0.7 ± 0.2	ND^c^	0.9 ± 0.2	ND^c^	1.2 ± 0.2	0.8 ± 0.1	0.9 ± 0.2	1.1 ± 0.1	0.9 ± 0.2	0.9 ± 0.3

a Two-tailed t-test p value < 0.05.

b Two-tailed t-test p value < 0.01.

c ND indicates the interactions were not determined in this assay, which examined relationships based on RCR network predictions.

Among the validated uncharacterized transcription factors, five out of six are activators. The overexpression of Wall-Associated AP2/ERF family protein, WAP1, encoded by *LOC_Os03g08470*, significantly activated *OsF5H1* (**Table 3**, **Figure 5**). To our knowledge, the only AP2/ERF protein previously experimentally demonstrated to function in cell wall regulation is SHINE2/WAX INDUCER (SHN2/WIN) (Ambavaram et al., 2011). WAP1 also has relatively high rank in the rice cell wall network of Hirano et al. (2013a). In addition to WAP1, the Wall-Associated basic Helix-Loop-helix family protein, WAHL1, encoded by *LOC_Os01g11910*, also significantly activated *OsF5H1* (**Table 3**, **Figure 5**).

Expression of the Wall-Associated Homeoomain protein, WAHD1, encoded by *LOC_Os12g43950*, significantly activated *OsCAD2* expression (**Table 3**, **Figure 5**). WADH1 is in the clade neighboring OsBLH6 (Jain et al., 2008), which is another bell-type homeodomain protein in the list of potential cell wall regulators (**Supplementary Table 5**), and which has been shown to activate lignin biosynthesis (Hirano et al., 2013b).

OsMYB13a, encoded by *LOC_Os02g41510*, and OsMYB13b, encoded by *LOC_Os04g43680*, both activated *Os4CL3* transcription. OsMYB13a also activated *OsCOMT1*, whereas OsMYB13b activated *OsCAD2* (**Table 3**, **Figure 5**). We named the two wall-associated R2R3 MYB proteins based on their ortholog in Arabidopsis, AtMYB13, which has not been associated with cell wall regulation to our knowledge.

We observed one repressor, Wall Associated C2H2, WACH1, encoded by *LOC_Os04g08060* (**Table 3**, **Figure 5**) in the protoplast assay. WACH1 repressed *Os4CL3* and the secondary cell wall-associated *OsCESA4*. The Arabidopsis ortholog is involved in stress responses (Ciftci-Yilmaz and Mittler, 2008). This protein also has relatively high rank in both the rice and Arabidopsis cell wall networks and in Hirano et al. (2013a).

## Discussion

The altered patterning and composition of grass cell walls compared with eudicots presents the need for regulatory innovation over the course of evolution. This work expands the systematic identification and experimental validation of transcription factors involved in grass cell wall synthesis regulation.

### RCRN Promotes Understanding of Rice Molecular Pathways

The RCRN shows greater genome coverage and quality compared to previous, publicly available rice gene networks. The heuristic approach for constructing the RCRN applied inverse mutual rank to the three original networks and then used a general linearized model to calibrate the co-expression network edges relative to the high-quality Bayesian comparative network, RiceNet v2. The slightly superior quality of the RCRN over even RiceNet v2 based on gene ontology similarity of connected nodes (**Figure 1**, **Supplementary Figure 1**) may be due to the observation that mutual rank improves reproducibility and overall performance of gene networks (Obayashi et al., 2014).

The RCRN also shows a lower false negative rate than RiceNet v2 based on the experimental gene expression network derived from characterizing the *myb61a-1* mutant line. RiceNet v2 and the RCRN predict 15 and 36 interactions between OsMYB61a and cell wall genes, respectively (**Supplementary Figure 10**). Compared to the gene expression measurements, RiceNet v2 and the RCRN have similar true positive rates of 40% (4 out of 9 validated interactions) and 39% (9 out 23 validated interactions), respectively. On the other hand, the networks differ in their relative false negative rate, which represents validated interactions in the gene expression analysis not predicted by the networks. The false negative percentage for RiceNet v2 is 53%, which is much higher than that of the RCRN, at 8.3%. In particular, RiceNet v2 misses interactions with BAHD-ATs and rice xylan biosynthesis genes (**Supplementary Figure 10**).

### Conservation and Divergence of Known Cell Wall Regulators in Angiosperms

The RCRN and the experimental evidence we report add to the literature to suggest that most orthologs of genetically verified secondary cell wall regulators maintain general functional conservation between grasses and eudicots but may differ in mechanistic details. In the RCRN, all rice orthologs of genetically tested Arabidopsis secondary cell wall regulators connect with cell wall-related genes (**Figure 2B**, **Supplementary Table 2**). Further, transient expression experiments with OsMYB58/63a indicate general conservation of function of this protein between rice and Arabidopsis (**Table 2**), in line with recent molecular genetic analysis in sorghum and switchgrass (Scully et al., 2016; Rao et al., 2019). Likewise, stable genetic and transient analyses in this study (**Figure 3**, **Supplementary Figure 9**, **Supplementary Table 2**) and the literature (Hirano et al., 2013b; Huang et al., 2015) suggest that the function of MYB61 in cell wall regulation is also broadly conserved. Taken together the data presented here add to a model that most secondary cell wall-associated transcription factors originated before the divergence of eudicots and monocots and have maintained similar functions during evolution (Rao et al., 2019).

Despite general conservation, our analyses support the notion that at least some of the molecular details of secondary cell wall regulation vary between rice and Arabidopsis (**Figure 2A**, **Supplementary Figure 2**). As network connectivity (node degree) reflects essentiality (Batada et al., 2006; He and Zhang, 2006; Yang et al., 2014), this metric suggests that several rice orthologs of known Arabidopsis cell wall regulators, especially OsVND6/7, may have altered importance relative to their roles in eudicots. This absence was observed previously, leading to the hypothesis that OsVND6/7 might have specialized to regulate other aspects of xylem differentiation in grasses (Hirano et al., 2013a). An alternative hypothesis, consistent with the activity of *Physcomitrella patens* VND7 homologs regulating secondary cell wall gene expression in Arabidopsis (Xu et al., 2014), is that NAC activity in rice is modulated by interactions with other proteins (e.g., Yamaguchi et al., 2010). The relatively low degree of the rice AtMYB46/83 ortholog was also surprising, given this protein’s orthologs’ important and conserved function in activating cell wall biosynthesis (Zhong et al., 2015). To our knowledge, this protein’s function has not been tested genetically in grasses, but we would predict that though its function in controlling secondary cell wall biosynthesis gene expression is retained, its role is diminished relative to the large number of other regulators that grasses utilize (Hirano et al., 2013a; Yan et al., 2013; Rao et al., 2019). While much remains to be elucidated, we speculate that differences in specific molecular interactions within the regulatory networks between grasses and eudicots may lead to variation in stem anatomy and secondary cell wall patterning between these lineages. Indeed, the literature that compares regulatory networks across species suggests that general conservation but subtle divergence across evolution might be more the rule than the exception. In another example, orthologs of the Arabidopsis stomatal initiation regulators also control stomatal development in *Brachypodium*, but the function of individual regulators and the relationships among them appear to differ (Raissig et al., 2016). Similarly, HOX genes regulate body-plan development in animals, but have evolved to also control abdomen pigmentation in some *Drosophila* species (Jeong et al., 2006; Rebeiz et al., 2009). Even within the grasses, ZmMYB31 and ZmMYB42 orthologs in rice and sorghum show distinct promoter occupancies and gene expression correlations *in vivo* (Agarwal et al., 2016).

### Incorporation of Grass-Expanded Genes Into Cell Wall Regulatory Networks

In contrast to cell pattern alterations, compositional differences between grasses and eudicots are better understood. Grass-specific cell wall synthesis enzymes fall into two classes, those with close homology to cell wall synthesis enzymes in eudicots (i.e., MLG synthesis) and those the close homologs of which appear to have other functions in eudicots (i.e., Mitchel clade BAHDs). We considered two models for evolution of regulation of these grass-specific cell wall synthesis genes: 1) that the grass-specific genes have been incorporated into conserved regulatory networks and 2) that grass-specific genes are regulated by novel regulators, not involved in cell wall synthesis regulation in other lineages. Our analysis supports the model that orthologs of known cell wall-associated transcription factors (i.e., OsMYB61a, OsMYB61b, and OsSND2) regulate grass-specific cell wall biosynthesis genes (**Table 2**, **Figures 4** and **5**).

Unsurprisingly, the differences among assays probing the function of OsMYB61a imply that there are additional regulators of grass-specific cell wall biosynthesis genes. Gene expression analyses in the *myb61a-1* mutant (**Figure 3**) and protoplast-based assays (**Table 2**) are consistent with OsMYB61a broadly regulating multiple classes of cell wall-related genes, including other regulators. This builds on previous results showing that OsMYB61a directly activates the promoters of rice *CESA4*, *CESA7*, and *CESA9* (Huang et al., 2015). However, even when OsMYB61a is capable of binding to a particular promoter, additional regulation is implicated. For example, the absence of expression changes of *OsCSLH1* with increased expression *OsMYB61a* and with DEX-induction of OsMYB61a-GR (**Figure 3C**) suggests that transcriptional repression of *OsCSLH1* might depend on other proteins absent in seedling-derived protoplasts, despite OsMYB61a*-OsCSLH1* promoter interaction capability (**Figure 3B**). Indeed, the modest cell wall compositional alterations (**Figure 4**), despite numerous gene expression changes observed in the *myb61a-1* mutant (**Figure 3**), are consistent with OsMYB61a controlling cell wall synthesis in concert with other regulators. This is consistent with the general architecture of cell wall regulation with many regulators binding both to the promoters of other transcription factors and directly to the promoters of cell wall enzymes (Taylor-Teeples et al., 2015).

### Cell Wall-Associated Transcription Factors

We provide experimental evidence for six previously unexamined transcription factors participating in cell wall regulation. Specifically, ectopic expression in protoplasts suggests that OsMYB13a, OsMYB13b, WAP1, WAHD1 and WAHL1 may activate lignin biosynthesis genes and that WACH1 may repress CESA and lignin biosynthesis genes (**Table 3**, **Figure 5**). Inparanoid analysis suggests that three out of six of the new wall-associated regulators have (co-)orthologs in Arabidopsis (**Supplementary Table 5**). Of these three, orthologs of MYB13a and WAHD1 are connected with known cell wall genes in the ACRN, suggesting that they are also likely to be wall-associated regulators in eudicots (**Supplementary Table 5**).

### Cell Wall-Associated Repressors and Applications

Besides identifying cell wall biosynthesis activators, this study uncovered two possible transcriptional repressors, OsSND2 and WACH1, which present both the opportunity to better understand the biology of cell wall patterning and to apply this to biotechnological biomass improvement. From a biological perspective, the role of negative regulators remains unclear. Their expression patterns tend to be largely similar to those of activators (**Supplementary Figures 5** and **8**) (Fornalé et al., 2006; Hussey et al., 2011; Shen et al., 2012), though negative correlations are apparent in some species (Agarwal et al., 2016). These proteins may function to repress expression in cells adjacent to those undergoing secondary cell wall synthesis, leading to tissue level patterning, or to moderate cell wall synthesis, halting the feed-forward loop that characterizes cell wall synthesis and other developmental events (Taylor-Teeples et al., 2015).

Especially as we learn more about the cellular mechanism for wall accumulation of components with roles in recalcitrance or as desirable bioproducts, regulation of transcriptional modules in a cell-type dependent fashion, as opposed to altering expression of single biosynthesis genes, may be more effective. At this point, WACH1, which is also present in Arabidopsis, is an attractive target for up-regulation to inhibit components of secondary cell wall synthesis, as has been demonstrated for switchgrass PvMYB4 (Shen et al., 2012; Baxter et al., 2015). SND2 may also be amenable for use as a negative modulator of secondary cell wall gene expression, though achieving this may require fine tuning. Indeed, SND2 was originally identified in Arabidopsis as a downstream target of SND1 and shown to be capable of activating transcription in yeast (Zhong et al., 2008). Zhong et al. (2008) also found that over expression of a dominant negative fusion protein showed thinner interfasicular fiber cell walls. On the other hand, over expression in Arabidopsis with a double *35S* promoter also decreased fiber cell wall thickness (Hussey et al., 2011), and *PvUbiquitin* promoter-controlled SND2-RNA interference in switchgrass resulted in marginal to no effects (Rao et al., 2019). Thus, SND2 activity may be sensitive to dosage and cellular context that alter its molecular partners as has been observed for other cell wall network regulators (Taylor-Teeples et al., 2015).

Finally, just as we have used the RCRN and ACRN to interrogate cell wall synthesis regulation, these high-quality networks should be useful for delineating other molecular pathways and their divergence between rice and Arabidopsis.

## Accession Numbers

Rice and Arabidopsis loci and nomenclature used in this work are listed in **Supplementary Tables 1** and **5**.

## Data Availability Statement

The RCRN and ACRN are available at: https://doi.org/10.5061/dryad.zgmsbcc69. Other datasets generated for this study are available provided with this manuscript or on request to the corresponding author.

## Author Contributions

KZ, LB, FL, SH, and K-HJ designed this study. KZ, FL, SR-G, H-JG, and PS performed the experiments. GA provided novel reagents. KZ, SR-G, and FL analyzed the data. KZ SR-G, SH, and LB wrote the manuscript and all authors approved the manuscript.

## Funding

This study was supported by the Department of Energy Plant Feedstock Genomics Program under grant No. DE-SC0006904 and by a grant from the Research Council of the University of Oklahoma Norman Campus to LB.

## Conflict of Interest

The authors declare that the research was conducted in the absence of any commercial or financial relationships that could be construed as a potential conflict of interest.
