# Supplementary material for: Rice Genome-Scale Network Integration Reveals Transcriptional Regulators of Grass Cell Wall Synthesis
Source: Front Plant Sci. 2019 Oct 18;10:1275. doi: 10.3389/fpls.2019.01275 (PMC6813959; doi:10.3389/fpls.2019.01275)
Supplement: Supplementary file 1 [file DataSheet_1.pdf]

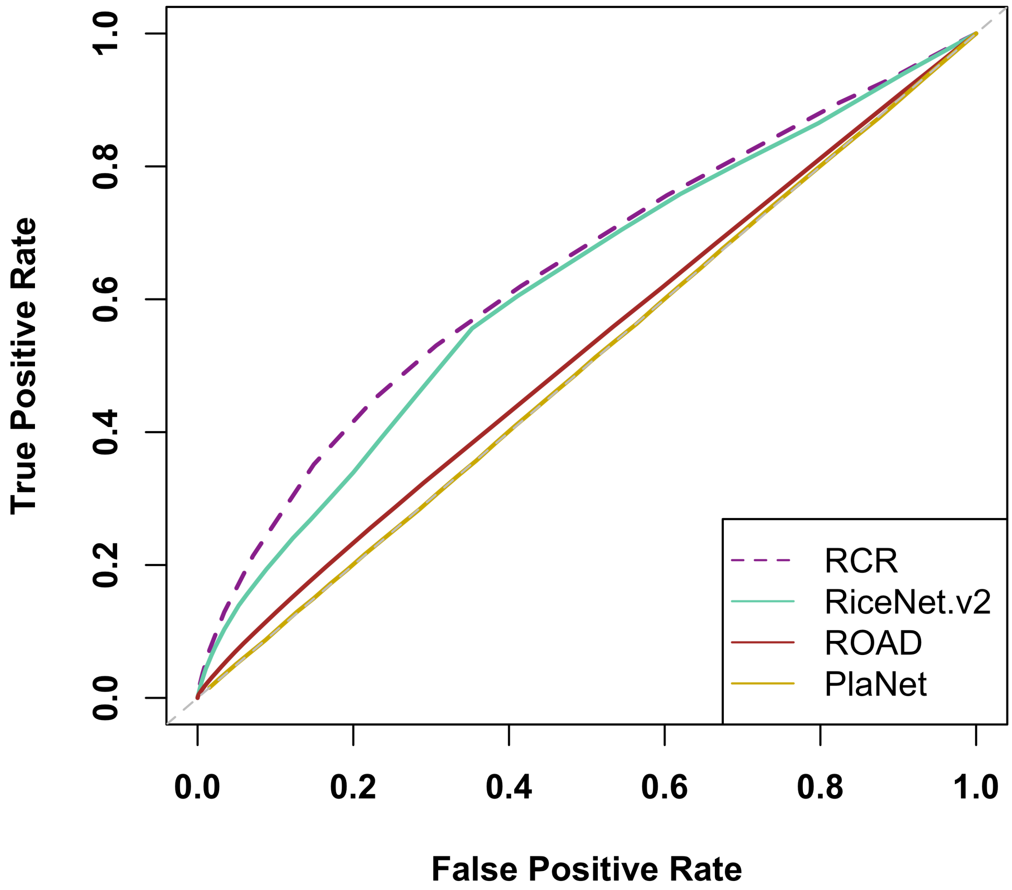

Supplementary Figure 1. The Receiver operating characteristic curve (ROC) for the rice networks. The ROC shows the True Positive Rate (TPR) vs. False Positive Rate (FPR) of Biological Process Gene Ontology terms as edge cut-off is decreased from left to right. For each network, forty different sub-networks were sampled different cut-offs analyzed in this study. False Positive Rate (FPR) = 1-specificity. The grey dashed diagonal represents random prediction.

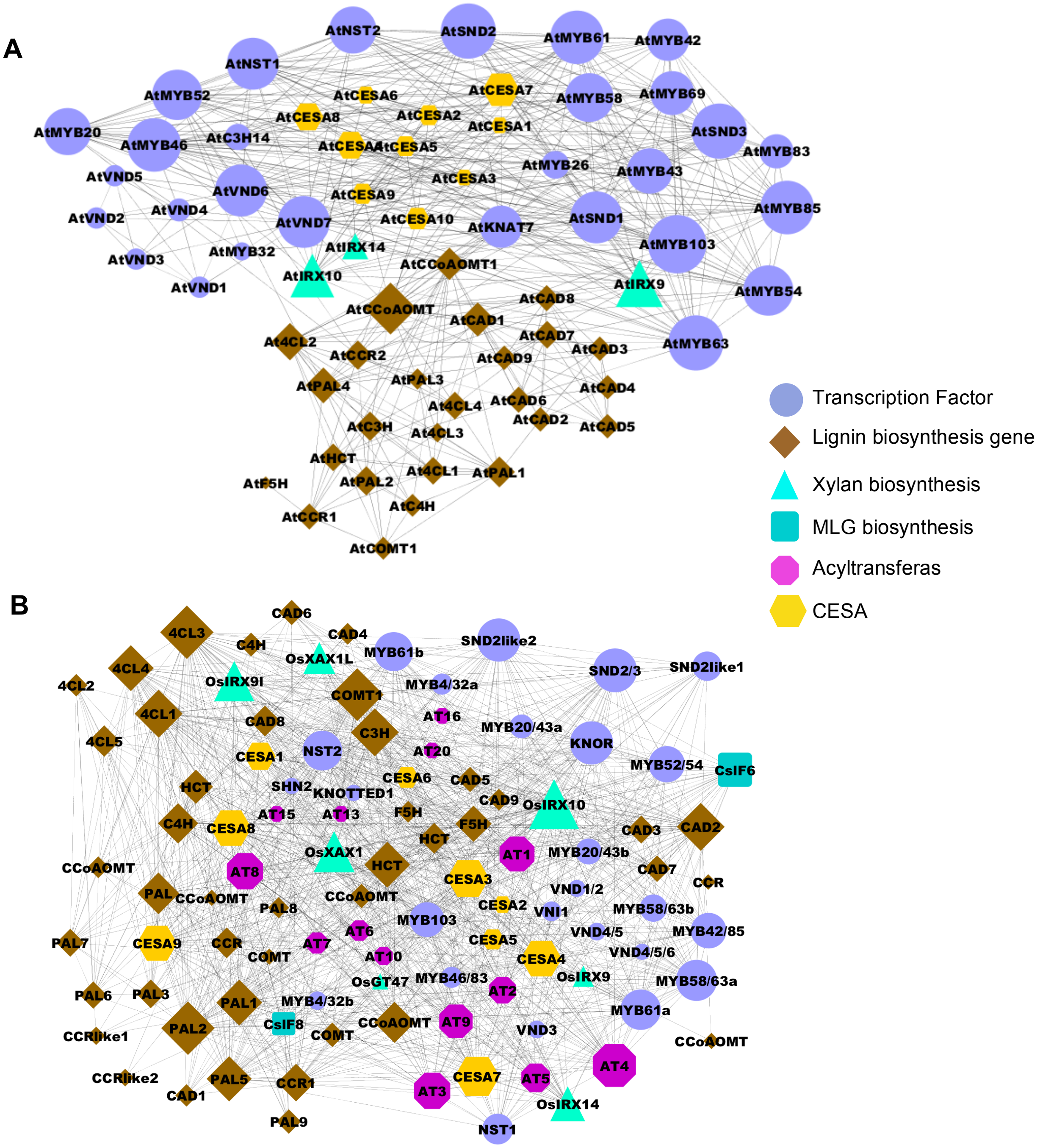

Supplementary Figure 2. Interactions among cell wall-related genes in A) Arabidopsis and B) rice based on combined networks. Colors and shapes represent gene families involved in cell wall biosynthesis and regulation as described in the key. The size of each node is proportional to the degree, i.e. number of connections with other cell wall-related genes. See Supplementary Table 1 for locus IDs and gene abbreviation explanations.

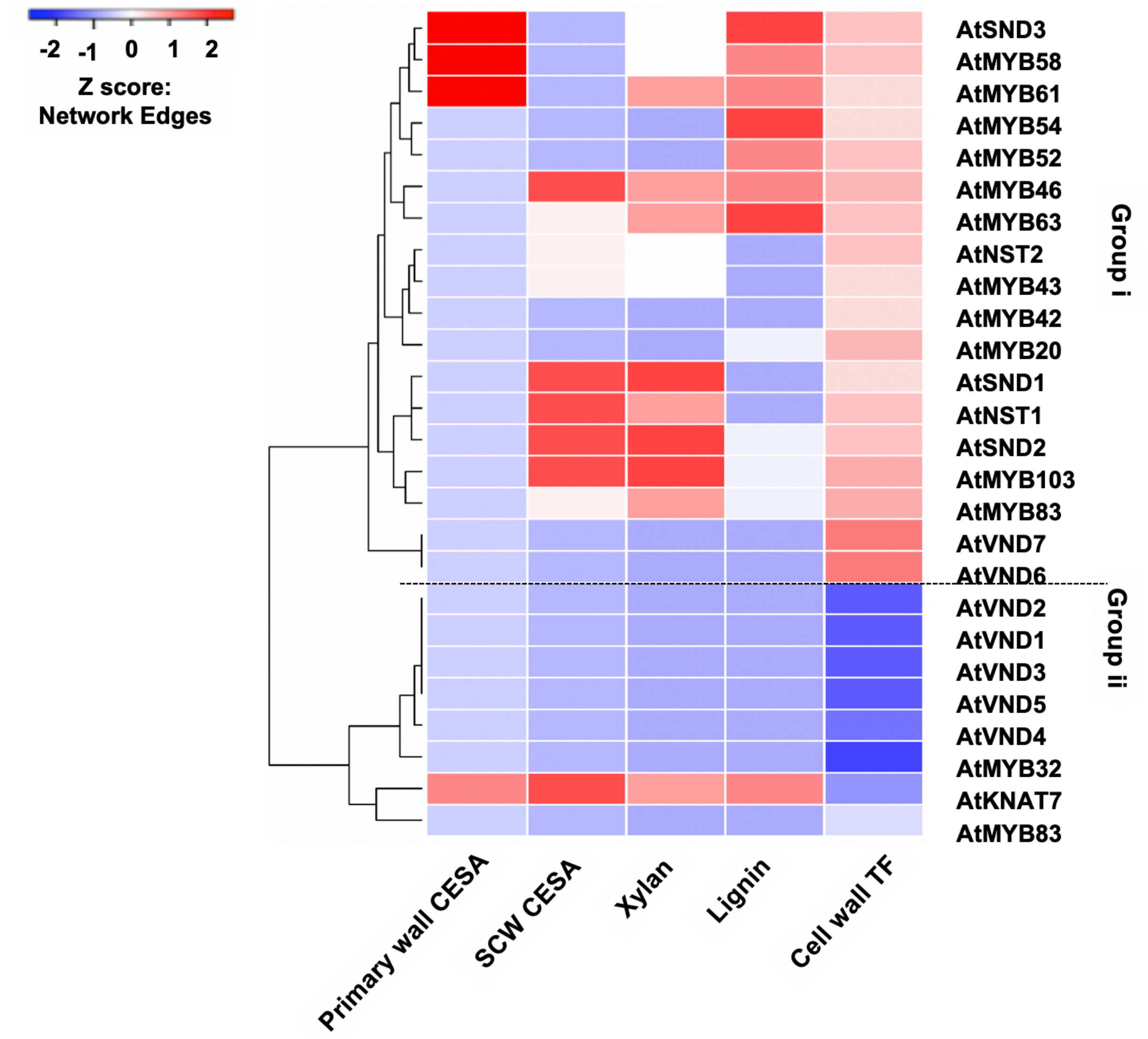

Supplementary Figure 3. Interactions between *Arabidopsis* cell wall transcription factors and different classes biosynthesis genes. The heatmap displays z-score normalization of the number of interactions with transcription factors for each group of cell wall-related genes, as extracted from the ACRN without edge cutoffs. Z-score was calculated for each column. CESA: genes encoding cellulose synthase; TF: transcription factors. Locus IDs are listed in Supplementary Table 1.

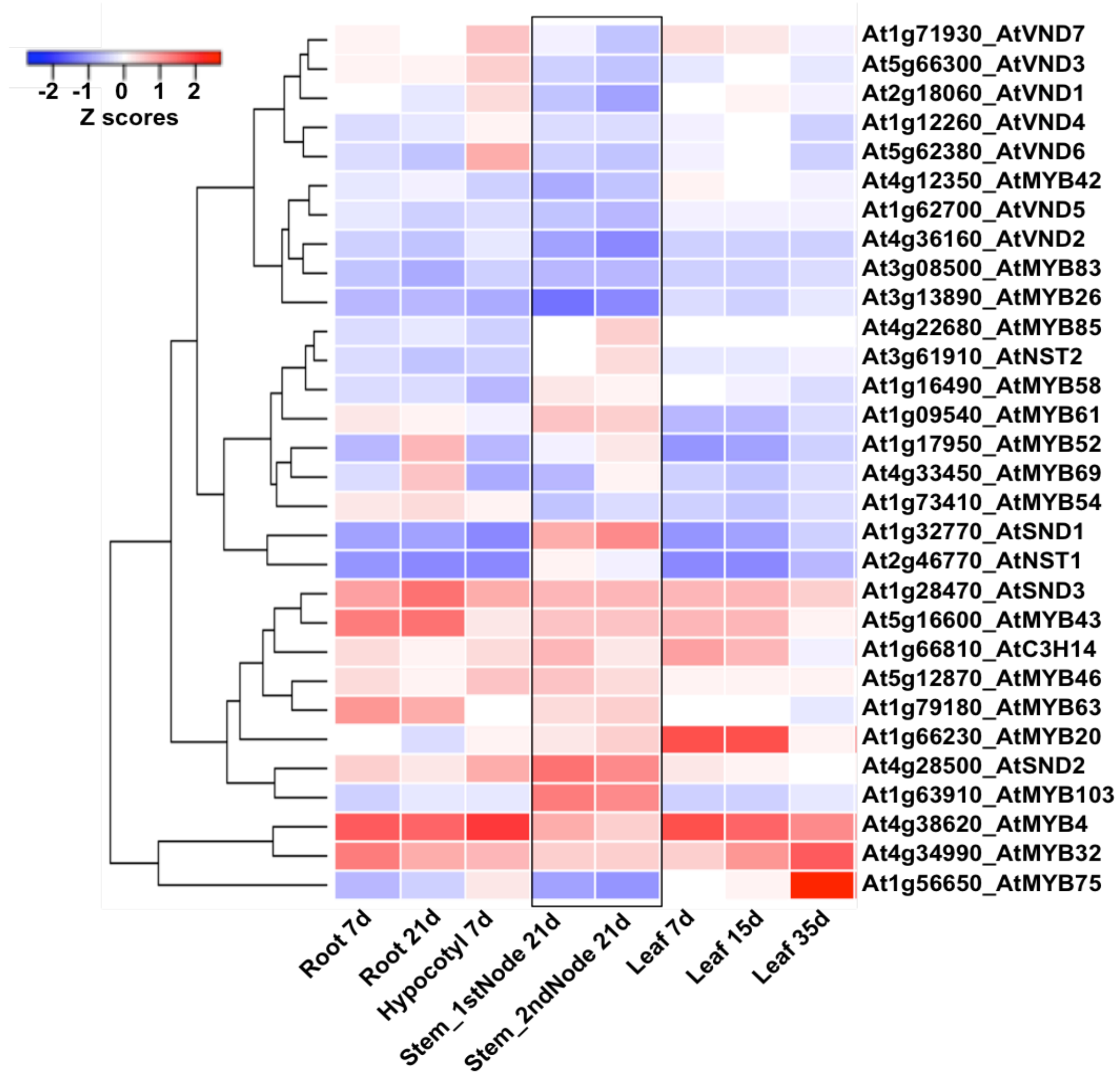

Supplementary Figure 4. Expression of *Arabidopsis* cell wall-associated transcription factors across development. The heatmap represents z-score normalized expression values for each tissue type. The data were extracted from the *Arabidopsis thaliana* expression atlas generated using Affymetrix microarrays (Schmid et al., 2005). The box highlights data for stems. “d” indicates days post germination.

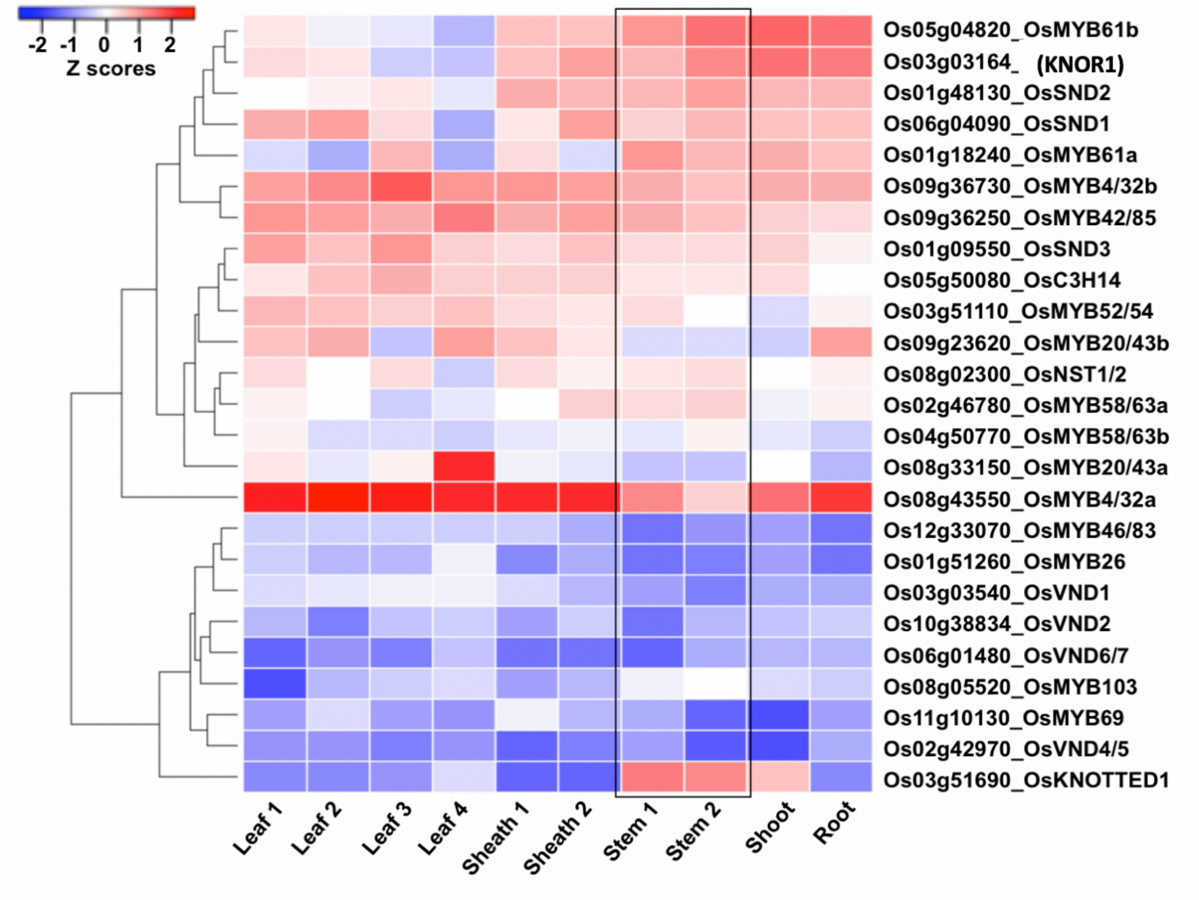

Supplementary Figure 5. Expression pattern of rice cell wall-related transcription factors across rice development. The heatmap represents z-score normalized expression values within each tissue type. The data were extracted from the rice expression atlas generated using Affymetrix rice microarrays (Wang et al., 2010). The box highlights data for stems.

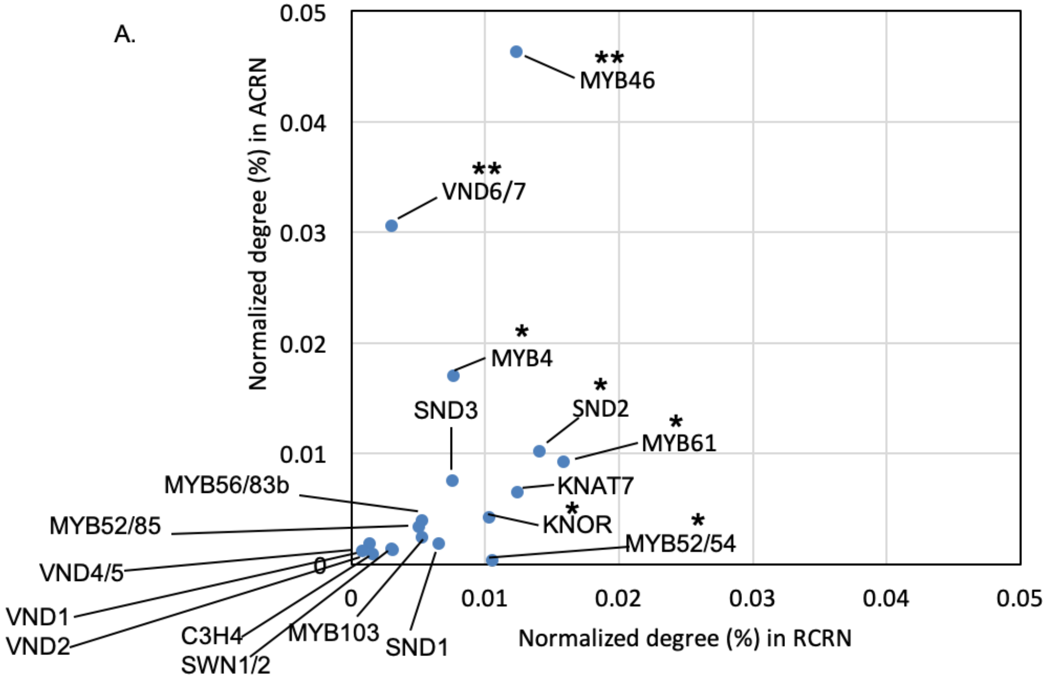

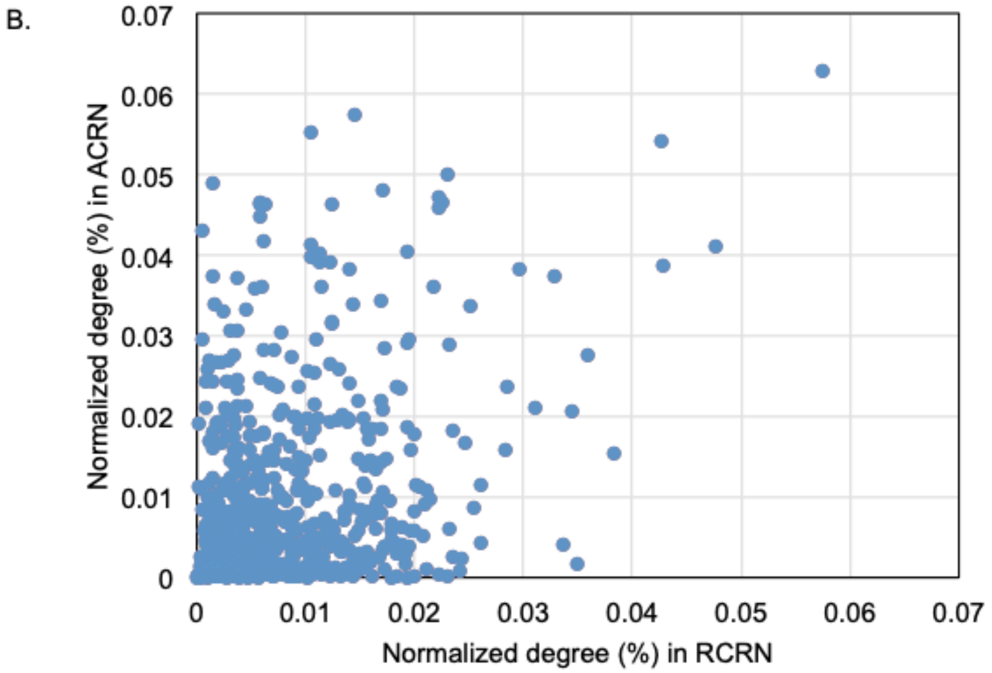

Supplementary Figure 6. Distribution of normalized degree for A) cell wall associated transcription factors and B) total transcription factors in the ACRN and RCRN. * indicates Fisher test p value < via 0.05. * *indicates Fisher test p value < 0.01. Interactions with all genes regardless of annotation edge scores are included.

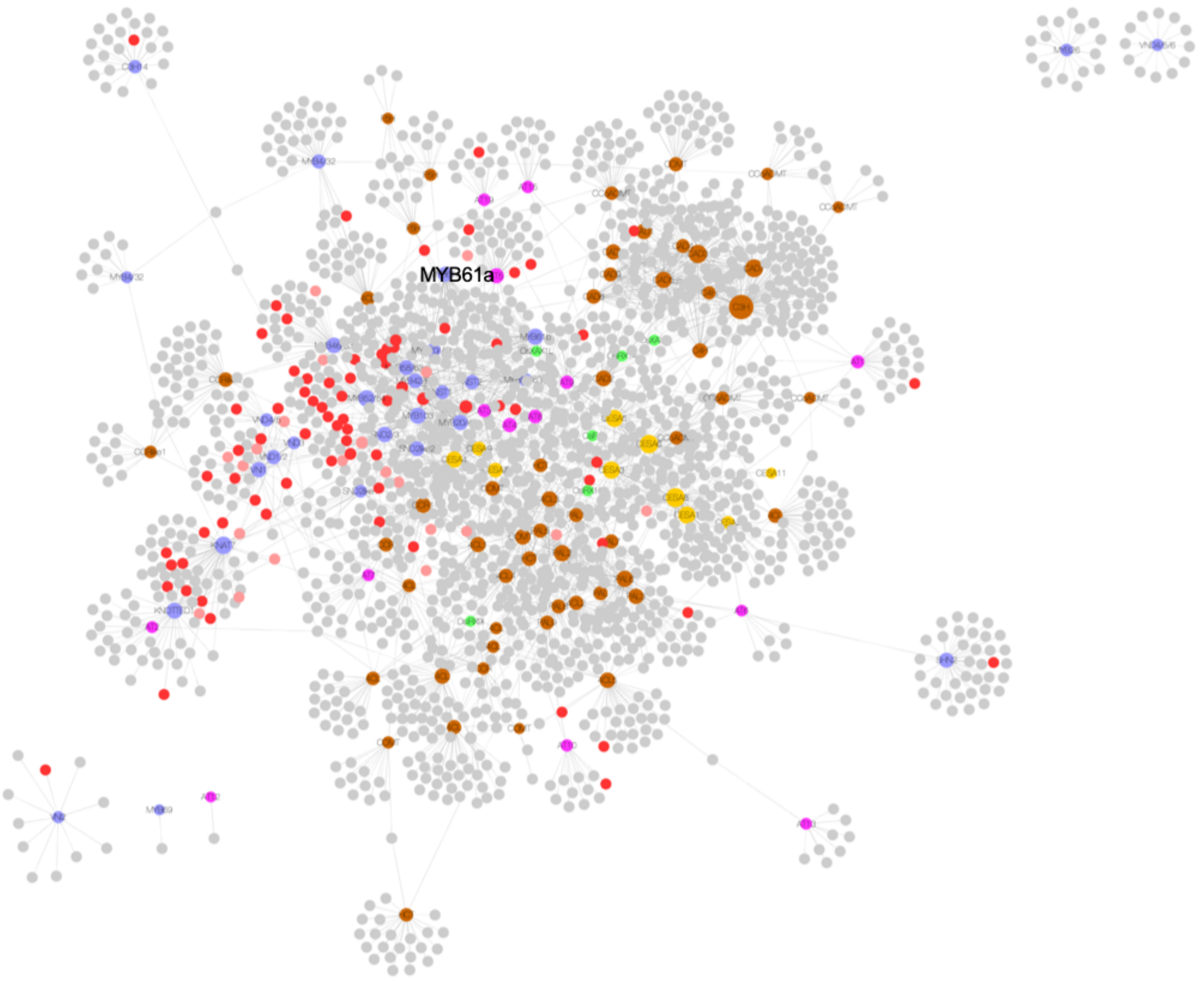

Supplementary Figure 7. Rice cell wall network with edge scores ≥ 0.03 within the RCRN. Cell wall genes and genes with direct connections to cell wall genes are shown. Blue: orthologs of known Arabidopsis cell wall-associated transcription factors. Magenta: BAHD acyltransferases. Brown: lignin biosynthesis genes. Green: hemicellulose biosynthesis genes. Yellow: cellulose biosynthesis genes. Pink: The 21 of the predicted 96 putative novel TFs that overlap with previously identified cell wall associated transcription factors in Hirano et al. 2013. Red: the rest of other putative novel TFs predicted in this study. Grey: genes with other annotations. The network was displayed using Cytoscape version 3.7.

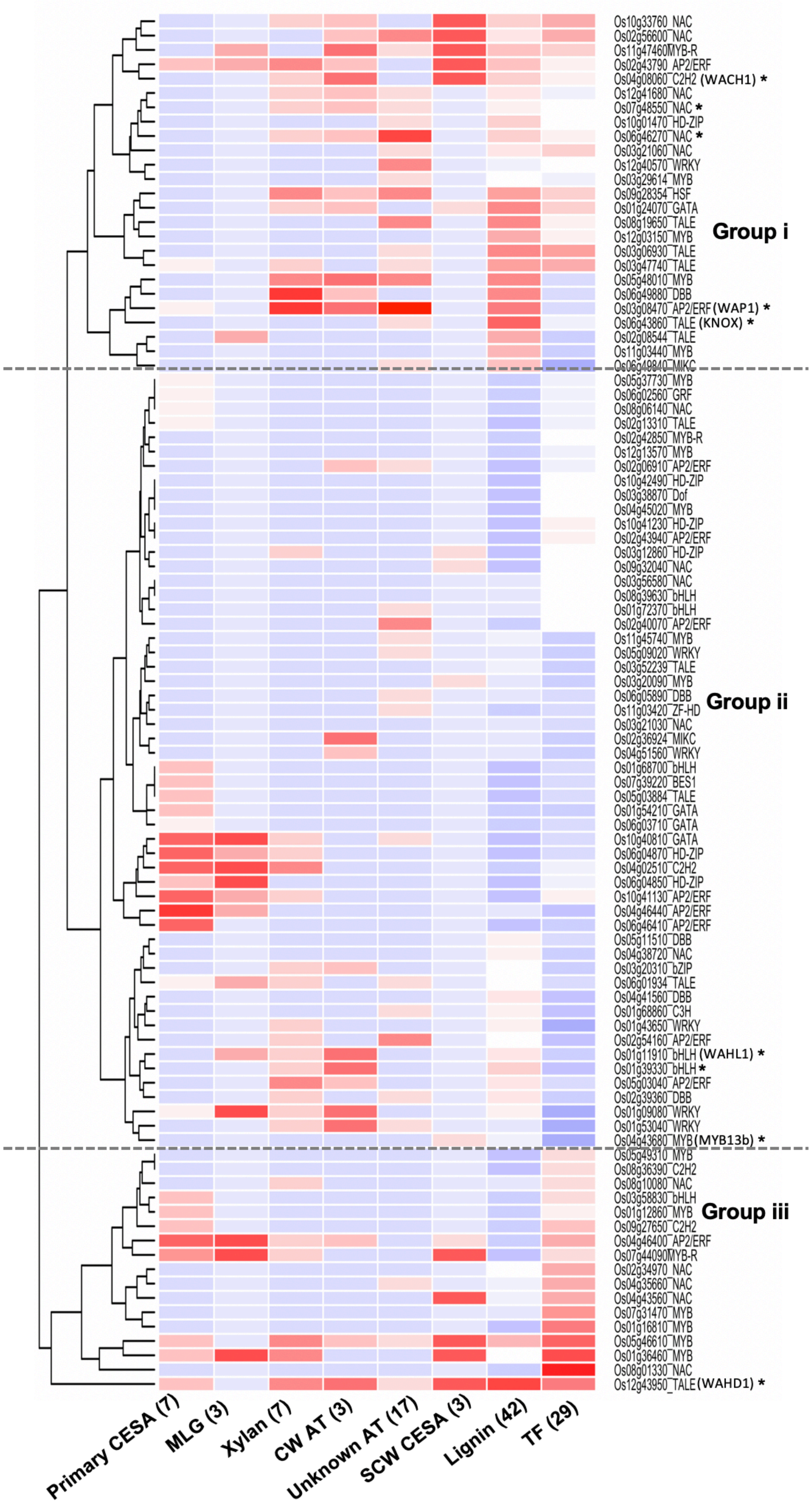
Os10g33760_NAC
Os02g56600_NAC
Os11g47460_MYB-R
Os02g43790_AP2/ERF
Os04g08060_C2H2 (WACH1) *
Os12g41680_NAC
Os07g48550_NAC *
Os10g01470_HD-ZIP
Os06g46270_NAC *
Os03g21060_NAC
Os12g40570_WRKY
Os03g29614_MYB
Os09g28354_HSF
Os01g24070_GATA
Os08g19650_TALE
Os12g03150_MYB
Os03g06930_TALE
Os03g47740_TALE
Os05g48010_MYB
Os06g49880_DBB
Os03g08470_AP2/ERF (WAP1) *
Os06g43860_TALE (KNOX) *
Os02g08544_TALE
Os11g03440_MYB
Os06g49840_MIKC
Group i
Os05g37730_MYB
Os06g02560_GRF
Os08g06140_NAC
Os02g13310_TALE
Os02g42850_MYB-R
Os12g13570_MYB
Os02g06910_AP2/ERF
Os10g42490_HD-ZIP
Os03g38870_Dof
Os04g45020_MYB
Os10g41230_HD-ZIP
Os02g43940_AP2/ERF
Os03g12860_HD-ZIP
Os09g32040_NAC
Os03g56580_NAC
Os08g39630_bHLH
Os01g72370_bHLH
Os02g40070_AP2/ERF
Os11g45740_MYB
Os05g09020_WRKY
Os03g52239_TALE
Os03g20090_MYB
Os06g05890_DBB
Os11g03420_ZF-HD
Os03g21030_NAC
Os02g36924_MIKC
Os04g51560_WRKY
Os01g68700_bHLH
Os07g39220_BES1
Os05g03884_TALE
Os01g54210_GATA
Os06g03710_GATA
Os10g40810_GATA
Os06g04870_HD-ZIP
Os04g02510_C2H2
Os06g04850_HD-ZIP
Os10g41130_AP2/ERF
Os04g46440_AP2/ERF
Os06g46410_AP2/ERF
Os05g11510_DBB
Os04g38720_NAC
Os03g20310_bZIP
Os06g01934_TALE
Os04g41560_DBB
Os01g68860_C3H
Os01g43650_WRKY
Os02g54160_AP2/ERF
Os01g11910_bHLH (WAHL1) *
Os01g39330_bHLH *
Os05g03040_AP2/ERF
Os02g39360_DBB
Os01g09080_WRKY
Os01g53040_WRKY
Os04g43680_MYB (MYB13b) *
Group ii
Os05g49310_MYB
Os08g36390_C2H2
Os08g10080_NAC
Os03g58830_bHLH
Os01g12860_MYB
Os09g27650_C2H2
Os04g46400_AP2/ERF
Os07g44090_MYB-R
Os02g34970_NAC
Os04g35660_NAC
Os04g43560_NAC
Os07g31470_MYB
Os01g16810_MYB
Os05g46610_MYB
Os01g36460_MYB
Os08g01330_NAC
Os12g43950_TALE (WAHD1) *
Group iii
Primary CESA (7)
MLG (3)
Xylan (7)
CW AT (3)
Unknown AT (17)
SCW CESA (3)
Lignin (42)
TF (29)

Supplementary Figure 8. Interactions between 96 high degree rice cell wall-associated transcription factors and various categories of cell wall-related genes in RCRN. The heatmap displays z-score normalization of the number of interactions with transcription factors for each group of cell wall-related genes, as extracted from the RCRN without edge cutoffs. The heatmap was generated using the heatmap.2 function in R. Asterisks represent transcription factors tested in protoplast-based transient assays. SCW: secondary cell wall.; CESA: genes encoding cellulose synthase; TF: transcription factors. Locus IDs are listed in Supplementary Table 5.

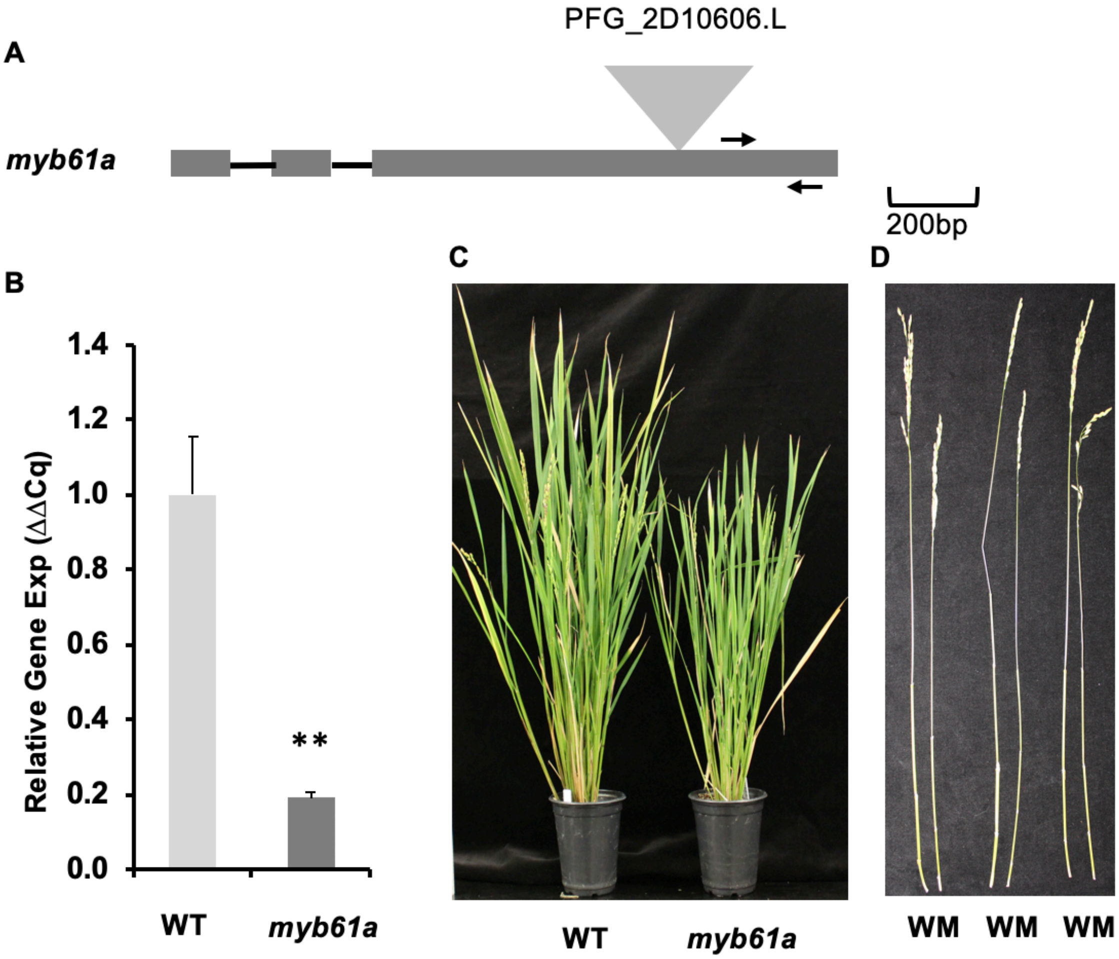

Supplementary Figure 9. Molecular characterization and stem phenotype of the *myb61a-1* line. (A) Insertion map of *myb61a-1* with the line number: PFG_2D10906.L. Arrows indicate the approximate position of primers used to measure the expression of Os*MYB61a*. (B) Fold change of *myb61a* in mutants (dark bar) comparing to the negative segregant, wild-type plants (WT, light bar). Five biological replicates of two-month old leaf samples were used. Error bars represent standard deviation. ** represents two-tailed t test p value < 0.01. (C) and (D) *myb61a* plants show dwarf phenotype and each internode from the mature tillers of the mutant (M) was shorter than that of the negative segregants (W). Representative, developmentally matched four-month old plants are shown.

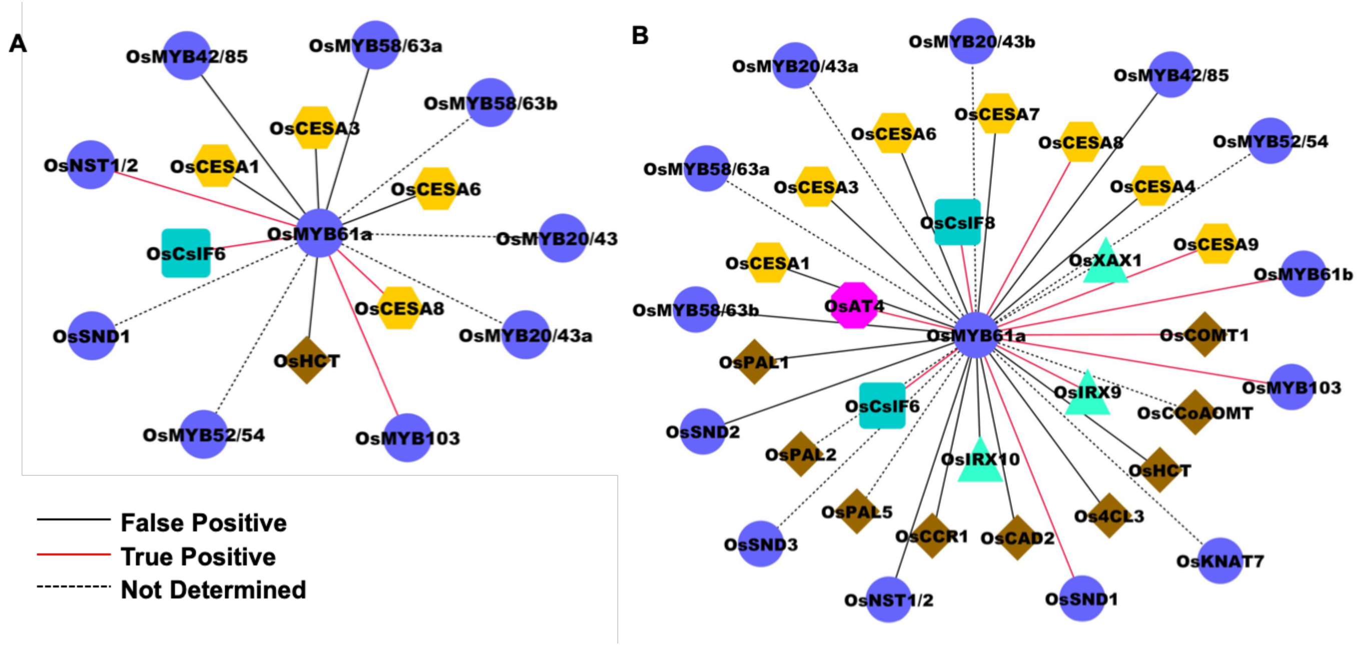

Supplementary Figure 10. The RCRN contains many more potential interactions between OsMYB61a and cell wall-related genes than RiceNet v2. A) RiceNet v2 OsMYB61a-cell wall network. B) RCRN OsMYB61a-cell wall network. True positive and false positive edges are based on gene expression with or without significant changes, respectively, within *myb61a-1* mutant plants compared to wild type, negative segregants. Not determined indicates expression of the gene was not tested.
