# Supplementary material for: Rice Genome-Scale Network Integration Reveals Transcriptional Regulators of Grass Cell Wall Synthesis
Source: Front Plant Sci. 2019 Oct 18;10:1275. doi: 10.3389/fpls.2019.01275 (PMC6813959; doi:10.3389/fpls.2019.01275)
Supplement: Supplementary file 3 [file Table_2.docx]

**Supplementary Table 2.** Recall of known interactions between transcription factors and cell wall biosynthesis genes in the RCR network.

| **Source** | **At TF-CW gene promoter interactions** ^a^ | **Orthologous pairs in rice** ^b^ | **Present in RCRN** ^c^ |
| --- | --- | --- | --- |
| Literature ^d^ | 134 | 105 | 97 |
| At root xylem Y1H ^e^ | 623 | 355 | 22 |
| Total | 757 | 460 | 119 |

^a^ *Arabidopsis thaliana* (At) transcription factor-cell wall gene promoter interactions reported in the literature.

^b^ Orthologous pairs indicates that both Arabidopsis gene products (transcription factor and cell wall target) are encoded in the rice genome based on Inparanoid analysis.

^c^ Present in RCRN indicates that the Rice Combined inverse Ranked Network possesses an edge for the pair without an edge value cut-off.

^d^ Based on a comprehensive analysis of the Arabidopsis cell wall regulation literature.

^e^ Yeast-one hybrid (Y1H) screen of Arabidopsis xylem secondary cell wall biosynthesis genes (Taylor-Teeples et al. 2015).
