# Supplementary material for: Rice Genome-Scale Network Integration Reveals Transcriptional Regulators of Grass Cell Wall Synthesis
Source: Front Plant Sci. 2019 Oct 18;10:1275. doi: 10.3389/fpls.2019.01275 (PMC6813959; doi:10.3389/fpls.2019.01275)
Supplement: Supplementary file 4 [file Table_3.docx]

**Supplementary Table 3.** Degree of transcription factors (TF) in Arabidopsis and rice based on connections with cell wall genes without cutoffs.

| TF | Degree based on cell wall genes | Size of subnetwork only with cell wall genes | Normalized degree (%) |
| --- | --- | --- | --- |
| AtKNAT7 | 19 | 550 | 3.45 |
| AtMYB103 | 29 | 550 | 5.27 |
| AtMYB20 | 22 | 550 | 4.00 |
| AtMYB32 | 4 | 550 | 0.73 |
| AtMYB42 | 19 | 550 | 3.45 |
| AtMYB43 | 21 | 550 | 3.82 |
| AtMYB46 | 26 | 550 | 4.73 |
| AtMYB52 | 24 | 550 | 4.36 |
| AtMYB54 | 24 | 550 | 4.36 |
| AtMYB58 | 23 | 550 | 4.18 |
| AtMYB63 | 27 | 550 | 4.91 |
| AtMYB83 | 14 | 550 | 2.55 |
| AtMYB85 | 26 | 550 | 4.73 |
| AtNST1 | 25 | 550 | 4.55 |
| AtNST2 | 22 | 550 | 4.00 |
| AtSND1 | 25 | 550 | 4.55 |
| AtSND2 | 27 | 550 | 4.91 |
| AtSND3 | 27 | 550 | 4.91 |
| AtVND1 | 6 | 550 | 1.09 |
| AtVND2 | 6 | 550 | 1.09 |
| AtVND3 | 6 | 550 | 1.09 |
| AtVND4 | 7 | 550 | 1.27 |
| AtVND5 | 6 | 550 | 1.09 |
| AtVND6 | 25 | 550 | 4.55 |
| AtVND7 | 25 | 550 | 4.55 |
| AtMYB61 | 26 | 550 | 4.73 |
| AtMYB4 | 5 | 550 | 0.91 |
| OsSND2 | 42 | 448 | 9.38 |
| KNOR1 | 41 | 448 | 9.15 |
| OsSND3 | 40 | 448 | 8.93 |
| OsMYB58/63a | 39 | 448 | 8.71 |
| OsMYB61a | 37 | 448 | 8.26 |
| OsNST2 | 36 | 448 | 8.04 |
| OsMYB61b | 35 | 448 | 7.81 |
| OsMYB52/54 | 32 | 448 | 7.14 |
| OsMYB42/85a | 30 | 448 | 6.70 |
| OsMYB103 | 29 | 448 | 6.47 |
| OsNST1 | 23 | 448 | 5.13 |
| OsSND1 | 21 | 448 | 4.69 |
| OsMYB58/63b | 18 | 448 | 4.02 |
| OsMYB20/43b | 16 | 448 | 3.57 |
| OsMYB20/43a | 13 | 448 | 2.90 |
| OsMYB4/32a | 9 | 448 | 2.01 |
| OsMYB46/83b | 9 | 448 | 2.01 |
| OsMYB4/32b | 5 | 448 | 1.12 |
| OsVND3 | 4 | 448 | 0.89 |
| OsVND1/2 | 3 | 448 | 0.67 |
| OsVND6/7 | 2 | 448 | 0.45 |
