# Supplementary material for: Rice Genome-Scale Network Integration Reveals Transcriptional Regulators of Grass Cell Wall Synthesis
Source: Front Plant Sci. 2019 Oct 18;10:1275. doi: 10.3389/fpls.2019.01275 (PMC6813959; doi:10.3389/fpls.2019.01275)
Supplement: Supplementary file 5 [file Table_4.docx]

**Supplemental Table 4**. Families of the 96 putative novel cell wall-associated transcription factors with 5 or more edges in the cell wall network extracted from the RCRN.

| TF Family | Total TF |
| --- | --- |
| MYB | 19 |
| NAC | 16 |
| TALE | 10 |
| AP2/ERF | 9 |
| HD-ZIP | 7 |
| bHLH | 6 |
| WRKY | 6 |
| DBB | 5 |
| C2H2 | 4 |
| GATA | 3 |
| ARF | 2 |
| MIKC | 2 |
| BES1 | 1 |
| bZIP | 1 |
| C3H | 1 |
| Dof | 1 |
| GRAS | 1 |
| GRF | 1 |
| HSF | 1 |
