# Supplementary material for: Rice Genome-Scale Network Integration Reveals Transcriptional Regulators of Grass Cell Wall Synthesis
Source: Front Plant Sci. 2019 Oct 18;10:1275. doi: 10.3389/fpls.2019.01275 (PMC6813959; doi:10.3389/fpls.2019.01275)
Supplement: Supplementary file 7 [file Table_6.docx]

**Supporting Table 6**. Primers for genotyping *myb61a-1* mutants and cloning transcription factors used in this analysis.

(A) Primers for genotyping the *myb61a-1* line.

| Line | Locus ID | 5' primer | 3' primer | primer pair for T-DNA::plant junction |
| --- | --- | --- | --- | --- |
| *2D-10906* | *Os01g18240* | AAACTGGGGGCATCACAGTG | TGCTGGAGCACCGTTCCAA | 5' primer / L0.5 |

**(B)** Primers for cloning rice transcription factors.

| MSU Locus ID | Name | 5' Primer sequence | 3' Primer sequence |
| --- | --- | --- | --- |
| *LOC_Os01g11910* | *WAHL1* | CACCATGGGCTCTGCTCCGTTCGG | CACCTTGATCTGCATGTCCTTTGG |
| *LOC_Os01g39330* | *bHLH* | CACCATGGATGAGGTGTGGTGCAG | CACTCACTAATCAGCAATCA |
| *LOC_Os03g08470* | *WAP1* | CACCATGTGTGGAGGCGCCATCCT | GCATCAATGGAGGAGTACA |
| *LOC_Os04g08060* | *WACH1* | CACCATGGGCGTCCAGGAGGAGG | TCTGTCGCTTGTGCTGCTAG |
| *LOC_Os06g43860* | *KNOX* | CACCATGGCGTTCCACTACCAGGAC | CTCCAACCACGTTCTACCAAG |
| *LOC_Os06g46270* | *NAC* | CACCTCGATCCAGCATCTCAAGG | TCAACTGAGTGAGTTCCACA |
| *LOC_Os07g48550* | *NAC* | CACCATGTCGGAGTCGGAGGTGTC | TCACCACAAACTCCATCATCA |
| *LOC_Os10g39030* | *BLH* | CACCCATCTCTCCTCTCCTCCACCT | CCCGTCAATCACATCAACCT |
| *LOC_Os12g43950* | *WAHD1* | CACCTGTCCTGCTGGAATCATCAA | TACAAATCCCATGCCCTTTC |
| *LOC_Os01g48130* | *OsSND2* | CTGCACGATCTCTCGTCTT | ATATACCTGCCCTGCCCTCT |
| *LOC_Os04g50770* | *OsMYB58a* | AGAGCAACACGAGCAAGGAG | TGGGGTTACTCGTGATGACA |
| *LOC_Os02g41510* | *OsMYB13a* | AGGAGGAAAGGTCGGCAATG | AAGAATAGTGGTGGTAAGAA |
| *LOC_Os04g43680* | *OsMYB13b* | GCAAGAGGAGCAGAGCAGTT | TGATTCGCTCATGGACACTC |
| *LOC_Os05g04820* | *OsMYB61b* | ATGGGGAGGCATTCTTGCTGCTAC | TAGCATTGCACCTAGATATGTTC |
| *LOC_Os01g18240* | *OsMYB61a* | TCTGCCATAAGCTTCCATC | TTCATGTGGTGCTCTGTTCC |
